# Enhanced excitability of cortical neurons in low-divalent solutions is primarily mediated by altered voltage-dependence of voltage-gated sodium channels

**DOI:** 10.7554/eLife.67914

**Published:** 2021-05-11

**Authors:** Briana J Martiszus, Timur Tsintsadze, Wenhan Chang, Stephen M Smith

**Affiliations:** 1 Section of Pulmonary & Critical Care Medicine, VA Portland Health Care SystemPortlandUnited States; 2 Department of Medicine, Division of Pulmonary & Critical Care Medicine, Oregon Health & Science UniversityPortlandUnited States; 3 Endocrine Research Unit, Veterans Affairs Medical Center and University of California, San FranciscoSan FranciscoUnited States; RIKENJapan; National Institute of Neurological Disorders and Stroke, National Institutes of HealthUnited States

**Keywords:** calcium, surface charge, VGSC, NALCN, CaSR, excitability, Mouse

## Abstract

Increasing extracellular [Ca2+] ([Ca2+]o) strongly decreases intrinsic excitability in neurons but the mechanism is unclear. By one hypothesis, [Ca2+]o screens surface charge, reducing voltage-gated sodium channel (VGSC) activation and by another [Ca2+]o activates Calcium-sensing receptor (CaSR) closing the sodium-leak channel (NALCN). Here we report that neocortical neurons from CaSR-deficient (Casr-/-) mice had more negative resting potentials and did not fire spontaneously in reduced divalent-containing solution (T0.2) in contrast with wild-type (WT). However, after setting membrane potential to −70 mV, T0.2 application similarly depolarized and increased action potential firing in Casr-/- and WT neurons. Enhanced activation of VGSCs was the dominant contributor to the depolarization and increase in excitability by T0.2 and occurred due to hyperpolarizing shifts in VGSC window currents. CaSR deletion depolarized VGSC window currents but did not affect NALCN activation. Regulation of VGSC gating by external divalents is the key mechanism mediating divalent-dependent changes in neocortical neuron excitability.

## Introduction

Excitable tissues are strongly regulated by extracellular [Ca^2+^] ([Ca^2+^]_o_) (Neher and Sakaba, 2008; Ma et al., 2014; Jackman and Regehr, 2017). Movement of extracellular Ca^2+^, through voltage-activated Ca^2+^ channels (VACC), to the intracellular space is central to many of these processes (Ma et al., 2012a; Nanou and Catterall, 2018). However, a distinct, extracellular mechanism that is independent of synaptic transmission also contributes to [Ca^2+^]_o_-dependent regulation of nerve and muscle function (Adrian and Gelfan, 1933; Weidmann, 1955; Frankenhaeuser, 1957; Frankenhaeuser and Hodgkin, 1957). Decreases in [Ca^2+^]_o_ and [Mg^2+^]_o_ substantially facilitate spontaneous and evoked action potential generation which represents increased intrinsic excitability (Weidmann, 1955; Frankenhaeuser, 1957; Frankenhaeuser and Hodgkin, 1957). In the brain, physiological neuronal activity decreases [Ca^2+^]_o_ (Nicholson et al., 1978) leading to further increases in action potential firing in neighboring neurons (Anderson et al., 2013). The firing patterns and computational properties of local circuits are impacted substantially by this positive feedback leading to changes in brain behaviors (Titley et al., 2019). Furthermore, under pathological conditions, larger decreases in [Ca^2+^]_o_ occur, resulting in even greater changes in circuit activity, and implicating [Ca^2+^]_o_-dependent excitability in the pathogenesis of brain injury (Ayata and Lauritzen, 2015).

Classical studies proposed that the mechanism underlying [Ca^2+^]_o_-dependent excitability centers on voltage-gated sodium channel (VGSC) sensitivity to extracellular Ca^2+^. Reduced [Ca^2+^]_o_ was proposed to shift the effective voltage-dependent gating of the sodium conductance in the hyperpolarizing direction by reducing the screening of local negative charges on the extracellular face of the membrane or channel by external Ca^2+^ (Frankenhaeuser and Hodgkin, 1957; Hille, 1968). This surface potential screening model accounted for [Ca^2+^]_o_-dependent excitability in nerves and muscle without a need for additional molecular players and was widely accepted (Hille, 2001), although direct binding of Ca^2+^ to the VGSC was also proposed as contributing (Armstrong and Cota, 1991). However, this theory was challenged by new data demonstrating that activation of the sodium leak channel (NALCN), a non-selective cation channel, by the intracellular proteins, UNC79 and UNC80 (Lu et al., 2009; Lu et al., 2010) was necessary for [Ca^2+^]_o_-dependent excitability to occur in hippocampal neurons. Following the deletion of NALCN or UNC79, [Ca^2+^]_o_-dependent excitability was completely lost suggesting the increased excitability resulted from the activation of the non-rectifying NALCN which depolarized neurons and increased the likelihood of action potential generation *independent* of changes in VGSC function (Lu et al., 2010). The calcium-sensing receptor (CaSR), a G-protein-coupled receptor (GPCR), was hypothesized to detect and transduce the [Ca^2+^]_o_ changes and signal to the downstream multistep pathway (Lu et al., 2010). CaSR is well-positioned as a candidate [Ca^2+^]_o_ detector because at nerve terminals it detects [Ca^2+^]_o_ and regulates a non-selective cation channel (Smith et al., 2004; Chen et al., 2010) and because it transduces changes in [Ca^2+^]_o_ into NALCN activity following heterologous co-expression of CaSR, NALCN, UNC79, and UNC80 (Lu et al., 2010). Interest in the UNC79-UNC80-NALCN pathway has also risen, due to its essential role in the maintenance of respiration (Lu et al., 2007), the regulation of circadian rhythms (Lear et al., 2013; Flourakis et al., 2015), and because mutations of UNC80 and NALCN cause neurodevelopmental disorders, characterized by development delay and hypotonia (Al-Sayed et al., 2013; Perez et al., 2016).

Here, we address the question of whether the G-protein mediated NALCN pathway or VGSCs transduce the [Ca^2+^]_o_-dependent effects on excitability. We test if CaSR is a modulator of neuronal excitability via its action on a nonselective cation channel, determine the impact of CaSR expression on factors of intrinsic neuronal excitability, and examine the relative contributions of [Ca^2+^]_o_-regulated changes on VGSC and NALCN gating. In recordings from neocortical neurons, isolated by pharmacological block of excitatory and inhibitory inputs, we determine that neuronal firing is increased by decreasing external divalent concentrations and that this is almost entirely attributable to [Ca^2+^]_o_-dependent shifts in VGSC gating. Surprisingly, CaSR deletion substantially shifted VGSC gating, but had no effect on NALCN sensitivity to [Ca^2+^]_o_. Taken together our experiments indicate that acute [Ca^2+^]_o_-dependent increases in neuronal excitability result from changes in VGSC and NALCN gating and that CaSR contributes by an, as yet, uncharacterized action on VGSCs.

## Results

### CaSR and divalent-dependent neuronal excitability

**Figure 1.**
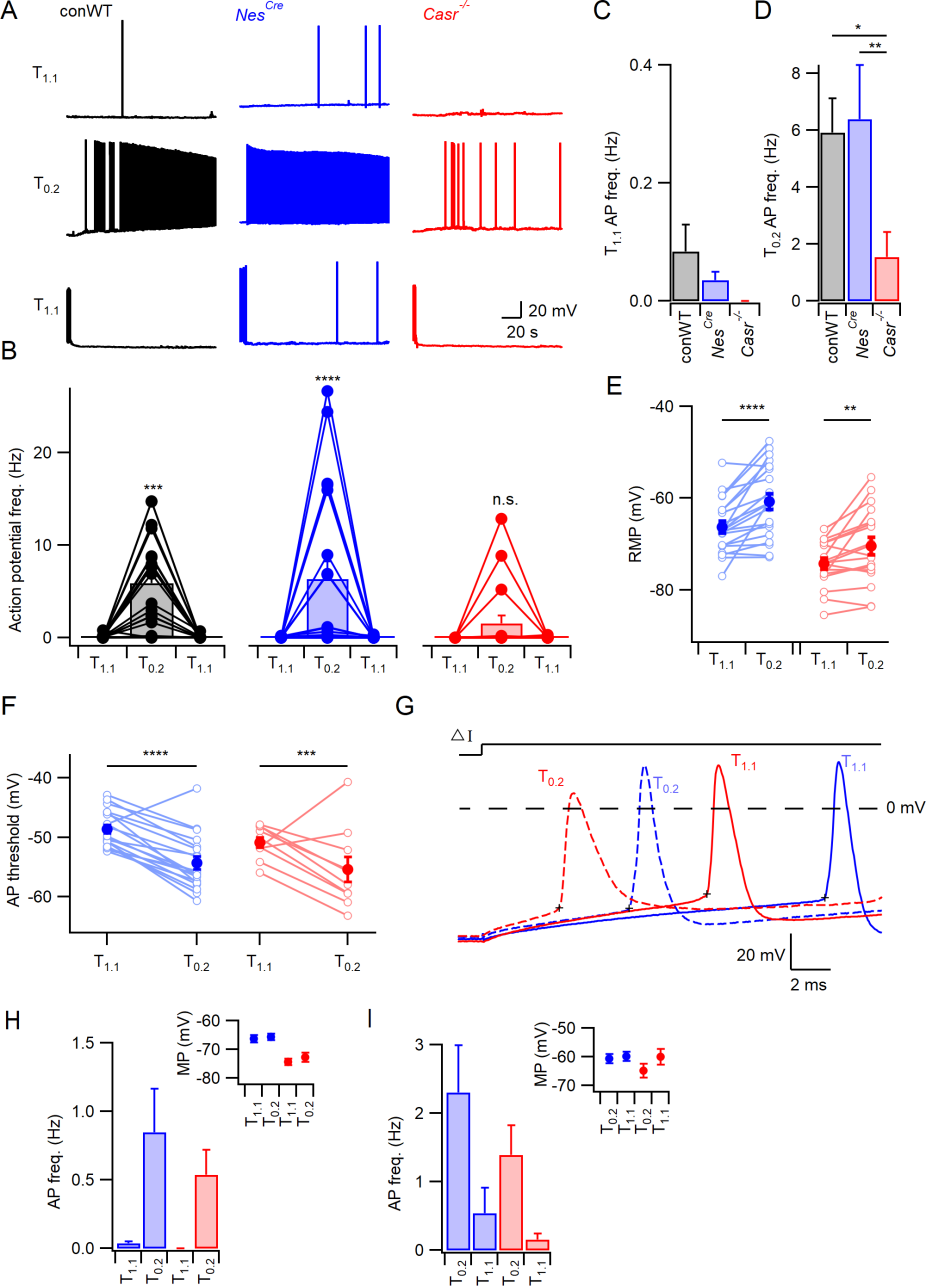
CaSR deletion reduces divalent-dependent excitability. (**A**) Spontaneous voltage traces at RMP following the application of solutions with different divalent concentrations (T_1_._1_ (upper traces), T_0.2_ (middle), and T_1.1_ recovery (lower)) recorded in three individual neurons with or without CaSR (conWT (black), *Nes^Cre^* (blue) and *Casr^-/-^* (red)). Each trace depicts 150 s of continuous acquisition. (**B**) Histograms of average action potential (AP) frequency (Hz) recorded using the same solutions: T_1.1_, T_0.2_, and T_1.1_ recovery. Individual recordings represented by open circles linked with lines and average is represented with a bar. From left to right graphs depict conWT (n = 18), *Nes^Cre^* (n = 21), and *Casr^-/-^* (n = 18). ANOVA: Post-hoc tests (Sidak compensated for multiple comparisons here and in all later figures) showed that action potential frequency increased in conWT (p=0.0009) and *Nes^Cre^* (P, 0.0001), but not *Casr^-/-^* (p=0.6697) neurons when changing from T_1.1_ to T_0.2_ (Figure 1—source data 1). (**C**) Baseline average action potential frequency in T_1.1_. was unaffected by genotype (p>0.999). (**D**) Average action potential frequency with T_0.2_ application was the same in conWT and *Nes^Cre^* (p=0.9831) and higher than in *Casr^-/-^* neurons (p=0.013 and 0.0033, respectively). (**E**) Plot of effect of external divalent concentration and CaSR on RMP. Two-way RM ANOVA indicates that increasing [Ca^2+^]_o_ (F (1, 37)=31.65, p<0.0001) and CaSR deletion (F (1, 37)=19.1, p<0.0001) hyperpolarized the RMP without an interaction (F (1, 37)=1.035, p=0.3155). Post-hoc testing indicated RMP was depolarized with the switch to T_0.2_ in both *Nes^Cre^* and *Casr^-/-^*neurons (p<0.0001 and p=0.0066 for 21 and 18 recordings respectively; Figure 1—source data 1). (**F**) Plot of average action potential threshold in T_1.1_ and T_0.2_ in *Nes^Cre^* and *Casr^-/-^* neurons elicited as per panel G. Two-way RM ANOVA indicates that reducing [Ca^2+^]_o_ hyperpolarized the action potential threshold (F (1, 27)=56.48, p<0.0001) but that genotype had no effect (F (1, 27)=2.284, p=0.1424). [Ca^2+^]_o_ was highly effective in both *Nes^Cre^* and *Casr^-/-^*neurons (p<0.0001 and p=0.0003 for 19 and 10 recordings, respectively). Individual neuron values are represented by open circles linked by lines and averages by filled circles. (**G**) Exemplar action potentials elicited by current injection in a *Nes^Cre^* (blue) and a *Casr^-/-^* neuon (red) in T_1.1_ (unbroken) and T_0.2_ (broken). Action potential threshold is indicated by +for the first action potential elicited by current injection (50–200 pA) under the same conditions as panel E. (**H**) Histogram summarizing effects of divalents on action potential frequency in *Nes^Cre^* and Casr^-/-^ neurons after a current injection to counter divalent-dependent depolarization following T_0.2_ application. Two-way RM ANOVA indicates that reducing [Ca^2+^]_o_ increases the action potential frequency (F (1, 35)=11.54, p=0.0017) and that this is significant in the *Nes^Cre^* but not *Casr^-/-^*neurons (p=0.0075 and 0.1555 for 21 and 16 recordings, respectively). Inset shows average membrane potential after the current injection. (**I**) Histogram summarizing effects of divalents on action potential frequency in *Nes^Cre^* and Casr^-/-^ neurons after current injection in T_1.1_ to depolarize membrane potential to value recorded in T_0.2_. Two-way RM ANOVA indicates that reducing [Ca^2+^]_o_ increases the action potential frequency (F (1, 35)=45.09, p=0.0004) and that this is significant in the *Nes^Cre^* but not *Casr^-/-^*neurons (p=0.0044 and 0.056 for 21 and 16 recordings, respectively). Inset shows average membrane potential after the current injection. Figure 1—source data 1.Action potential frequency and resting membrane potential in conventional WT, *Nes^Cre^* and *Casr^-/-^* neurons in T_1.1_ or T_0.2_ with no current injection.RMP units are mV and each sub-column represents measurements from a single neuron.

Increased excitability following the reduction of [Ca^2+^]_o_ ([Ca^2+^]_o_-dependent excitability) was eliminated by deletion of UNC79 or NALCN in neurons, challenging the long-standing hypothesis that local or diffuse surface charge screening of VGSCs mediated these effects (Lu et al., 2010). But how were changes in external divalent ion concentrations transduced to UNC79 and NALCN? We tested if CaSR provided the link, by comparing excitability in wild-type (WT) and nestin Cre-recombinase expressing CaSR null-mutant (Nes^Cre^Casr^fl/fl^ abbreviated as *Casr^-/-^*) neurons that were genotyped by PCR (see Materials and methods; Chang et al., 2008). Quantification by RT-qPCR indicated >98% reduction in the *Casr* expression levels in neocortical cultures produced from *Casr^-/-^* mice compared to Cre-positive WT (*Nes^Cre^*; Figure 1—figure supplement 1). Current clamp recordings were performed to measure the intrinsic, spontaneous action potential firing rate from cultured, neocortical neurons. The cells were also pharmacologically isolated to prevent the confounding influence on action potential firing of changes in synaptic transmission following alterations of [Ca^2+^]_o_ and [Mg^2+^]_o_ (glutamatergic and GABAergic activity blocked by 10 µM CNQX, 50 µM APV, and 10 µM Gabazine). After establishing the whole-cell configuration, we measured the spontaneous action potential firing rates of conventional WT (conWT), *Nes^Cre^*, and *Casr^-/-^* neurons in physiological Tyrode solution (T_1.1_; containing 1.1 mM) at the resting membrane potential (RMP) and then in reduced Ca^2+^- and Mg^2+^-containing Tyrode (T_0.2_; containing 0.2 mM [Ca^2+^] and [Mg^2+^]). CaSR and sodium conductance gating are both sensitive to Ca^2+^ and Mg^2+^, with Ca^2+^ being two to three times more potent in both processes (Frankenhaeuser and Hodgkin, 1957; Brown et al., 1993). Consequently, we modified the concentrations of both divalents to utilize a greater fraction of the dynamic range of the phenomenon under study. The reduction in [Ca^2+^]_o_ and [Mg^2+^]_o_ caused an increase in spontaneous action potential firing in both types of WT (conventional and *Nes^Cre^*) neurons within 15 s of the solution change that was substantially attenuated in the *Casr^-/-^* neuron (Figure 1A, middle row). This divalent-dependent neuronal excitability was reversed within 10 s by changing the bath solution back to physiological external divalent concentrations (Figure 1A, lower row). The pooled data from repeat experiments indicated that on average the conWT and *Nes^Cre^* neurons were equally sensitive to decreased extracellular divalent concentration and had similarly low spontaneous basal levels of activity (<0.1 Hz, Figure 1B–D). Two-way repeated measures (RM) ANOVA confirmed a significant interaction indicating the response to changes of external divalent concentration were dependent on genotype (F (2,54)=3.193, p=0.049, Table 1). Post-hoc tests confirmed that the reduction in [Ca^2+^]_o_ and [Mg^2+^]_o_ substantially increased action potential frequency in conWT and *Nes^Cre^* but not *Casr^-/-^* neurons (Sidak compensated for multiple comparisons here and in all later tests, Figure 1B, p=0.0009,<0.0001, and = 0.6697, respectively). Having confirmed that the conWT and *Nes^Cre^* neurons responded quantitatively the same to decreases in external divalents we used *Nes^Cre^* neurons alone as controls in subsequent experiments examining CaSR function. These data indicate that CaSR deletion substantially attenuates the increase in spontaneous firing at the RMP produced by reductions in external divalent concentrations in neurons.

**Figure 1—figure supplement 1.**
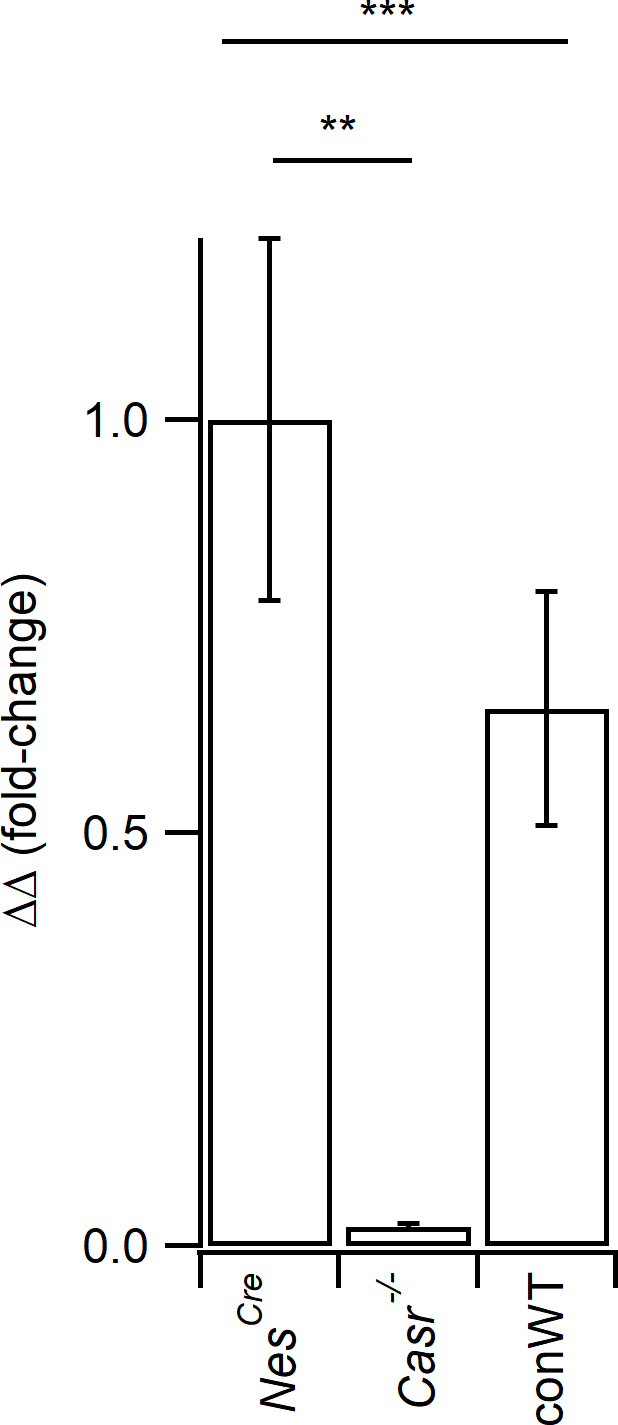
Casr expression levels reduced in *Casr^-/-^* neurons. Casr expression levels shown as delta delta with actin used as normalizing gene. Average values of 1.0, 0.02, and 0.65 for *Nes^Cre^*, *Casr^-/-^*, and conWT respectively with each genotype reflecting the average data from six cultures each represented by triplicate samples. The Kruskal-Wallis test indicates differences between genotypes (p=0.0002) with Dunn’s multiple comparison showing Casr expression levels are lower in *Casr^-/-^* (p=0.0016), but not conWT (p=0.7739), than in *Nes^Cre^* neocortical cultures.

**Figure 1—figure supplement 2.**
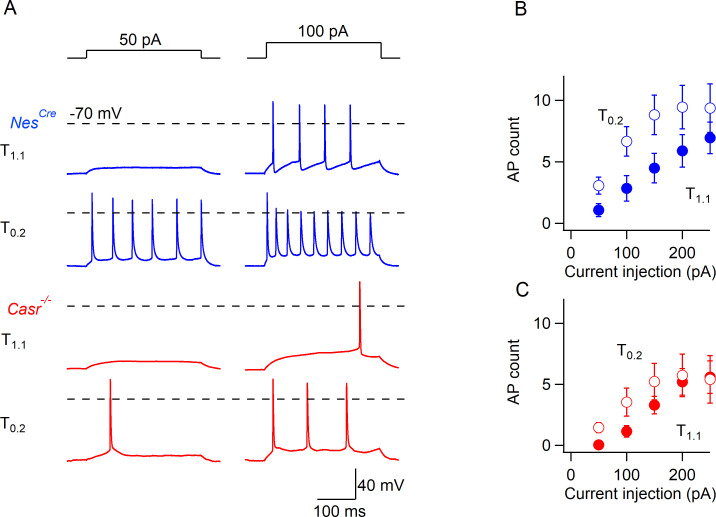
CaSR deletion reduces divalent-dependent excitability following the generation of action potentials elicited by current injections. (**A**) The neurons were held at RMP in T_1.1_ (zero basal current injection) or at the same potential in T_0.2_ (hyperpolarizing current injection as described in Figure 1H). The current injection is shown in the upper row. The broken horizontal line denotes 0 mV. Reduced divalent concentrations T_0.2_ increased action potentials in *Nes^Cre^* and *Casr^-/-^* neurons. (**B**) Action potentials were plotted versus the current injection for *Nes^Cre^* neurons (n = 19). (**C**) Action potentials were plotted versus the current injection for *Casr^-/-^* neurons (n = 11).

**Table 1.** Action potential frequency.

ANOVA table	SS	DF	MS	F (DFn, DFd)	P value
Interaction	130.0	2	64.99	F (2, 54)=3.193	p=0.0489
[Ca^2+^]_o_ on AP count	594.4	1	594.4	F (1, 54)=29.21	p<0.0001
Genotype	136.1	2	68.04	F (2, 54)=3.368	p=0.0418
Subjects (matching)	1091	54	20.20	F (54, 54)=0.9925	p=0.5110
Residual	1099	54	20.35		

### Does CaSR modulate RMP and divalent-dependent depolarization?

If CaSR-mediated NALCN-dependent depolarization is sufficient to account for the response to external divalent reduction, then *Nes^Cre^*, but not *Casr^-/-^*, neurons should depolarize in response to the switch to T_0.2_. However, the presence of CaSR and external divalent concentrations were both significant determinants of RMP (zero current injection; two-way RM ANOVA, Table 2, F (1,37)=19.1, p<0.0001 and F (1,37)=31.65, p<0.0001, respectively). In fact, the RMP of *Nes^Cre^* and *Casr^-/-^* neurons both depolarized similarly (Figure 1E; 5.6 ± 1.1 mV, p<0.0001 and 3.9 ± 1.2 mV, p=0.0066 respectively) when T_0.2_ was applied indicating the existence of a divalent-sensitive pathway in *Casr^-/-^* neurons.

**Table 2.** RMP.

ANOVA table	SS	DF	MS	F (DFn, DFd)	P value
Interaction	14.36	1	14.36	F (1, 37)=1.035	p=0.3155
[Ca^2+^]_o_ on RMP	438.9	1	438.9	F (1, 37)=31.65	p<0.0001
Genotype	1513	1	1513	F (1, 37)=19.10	p<0.0001
Subjects (matching)	2930	37	79.19	F (37, 37)=5.710	p<0.0001
Residual	513.2	37	13.87		

### Divalent-dependent firing persists after hyperpolarization

If NALCN-dependent depolarization is entirely responsible for the extracellular divalent-sensitive changes in neuronal excitability then reversal of this depolarization should prevent (or block) the increase in excitability seen in T_0.2_. To test this prediction, we measured spontaneous action potential frequency in T_0.2_ after adjusting the membrane potential to match the RMP observed in T_1.1_ (current injected to match the membrane potential was unique for each neuron). Action potential frequency in T_0.2_ was reduced by the hyperpolarization, but neurons remained sensitive to reduced divalent concentrations, although not CaSR deletion, indicating mechanisms besides NALCN were involved (Figure 1H, Table 3; F (1,35)=11.54, p=0.0017, 2-way RM ANOVA). Similarly, in the reciprocal experiment in which the membrane potential in T_1.1_ was depolarized to match that measured at low divalent concentration, the decrease in external divalent concentration increased action potential frequency (Figure 1I, Table 4; F (1,35)=15.17, p=0.0004, two-way RM ANOVA), and this was significant in *Nes^Cre^* but not *Casr^-/-^* neurons (Figure 1I, p=0.004). Ineffective matching of the membrane potential following solution changes did not account for the persistence of divalent-dependent excitability (insets, Figure 1H,I). The sustained sensitivity of spontaneous firing to reduced external divalent concentrations following hyperpolarization of the membrane potential indicated another mechanism, other than NALCN-mediated depolarization, was contributing to the extracellular divalent-sensitive changes in neuronal excitability. Divalent-dependent excitability was also evident in response to transient depolarizing currents (300 ms), with T_0.2_ increasing action potential count over a range of current injections in *Nes^Cre^*, and to a lesser degree in *Casr^-/-^* neurons (Figure 1—figure supplement 2). This was observed despite hyperpolarization of the neuron while in T_0.2_ to the resting membrane potential measured in T_1.1_, consistent with it occurring independent of any NALCN-mediated depolarization.

**Table 3.** Action potential frequency.

ANOVA table	SS	DF	MS	F (DFn, DFd)	p Value
Interaction	0.3407	1	0.3407	F (1, 35)=0.4758	p=0.4949
[Ca^2+^]_o_ at hyperpolarizing injection	8.262	1	8.262	F (1, 35)=11.54	p=0.0017
Genotype	0.5380	1	0.5380	F (1, 35)=0.7309	p=0.3984
Subjects (matching)	25.76	35	0.7360	F (35, 35)=1.028	p=0.4679
Residual	25.06	35	0.7161		

**Table 4.** Action potential frequency.

ANOVA table	SS	DF	MS	F (DFn, DFd)	p Value
Interaction	0.6090	1	0.6090	F (1, 35)=0.2048	p=0.6536
[Ca^2+^]_o_ at depolarizing injection	45.09	1	45.09	F (1, 35)=15.17	p=0.0004
Genotype	5.982	1	5.982	F (1, 35)=0.9959	p=0.3252
Subjects (matching)	210.2	35	6.006	F (35, 35)=2.020	p=0.0204
Residual	104.1	35	2.973		

The action potential threshold was measured to determine if there was a difference in the apparent excitability of *Nes^Cre^* and *Casr^-/-^* neurons. Action potentials were elicited in T_1.1_ and T_0.2_ using minimal current injection (50–250 pA) and the threshold measured as the point at which dV/dt reached 20 mV/ms (Figure 1G, membrane potential-corrected as in Figure 1H to minimize the effect of the depolarization itself). The action potential threshold was hyperpolarized from −48.6 ± 0.7 mV to −54.3 ± 1.1 mV with the switch from T_1.1_ to T_0.2_ in *Nes^Cre^* neurons (Figure 1F) which would have increased excitability. However, the same effect was observed in *Casr^-/-^* neurons (−50.9 ± 0.86 mV to −55.4 ± 2.1 mV; F (1,27)=56.48, p<0.0001, two-way RM ANOVA,Table 5). As CaSR deletion did not affect action potential threshold under these conditions (Figure 1F), spike generation presumably occurred more frequently in the *Nes^Cre^* neurons due to the relatively depolarized membrane potential (8 mV positive than *Casr^-/-^* neurons, Figure 1E). The lack of effect of CaSR on spike threshold (F (1, 27)=2.284, p=0.142) in these experiments, indicated the reduced divalent sensitivity of *Casr^-/-^* (Figure 1E,F) was not simply due to altered action potential threshold.

**Table 5.** Action potential threshold.

ANOVA table	SS	DF	MS	F (DFn, DFd)	p Value
Interaction	0.5225	1	0.5225	F (1, 27)=0.07478	p=0.7866
[Ca^2+^]_o_ on AP threshold	394.6	1	394.6	F (1, 27)=56.48	p<0.0001
Genotype	54.34	1	54.34	F (1, 27)=2.284	p=0.1424
Subjects (matching)	642.5	27	23.80	F (27, 27)=3.406	p=0.0011
Residual	188.6	27	6.987		

Overall these data support the idea that CaSR played a role in mediating divalent dependent changes in excitability, but that neurons also possessed CaSR-independent mechanisms to fully account for the divalent-dependent excitability.

### CaSR effects on divalent-dependent excitability attenuated by matching membrane potential

**Figure 2.**
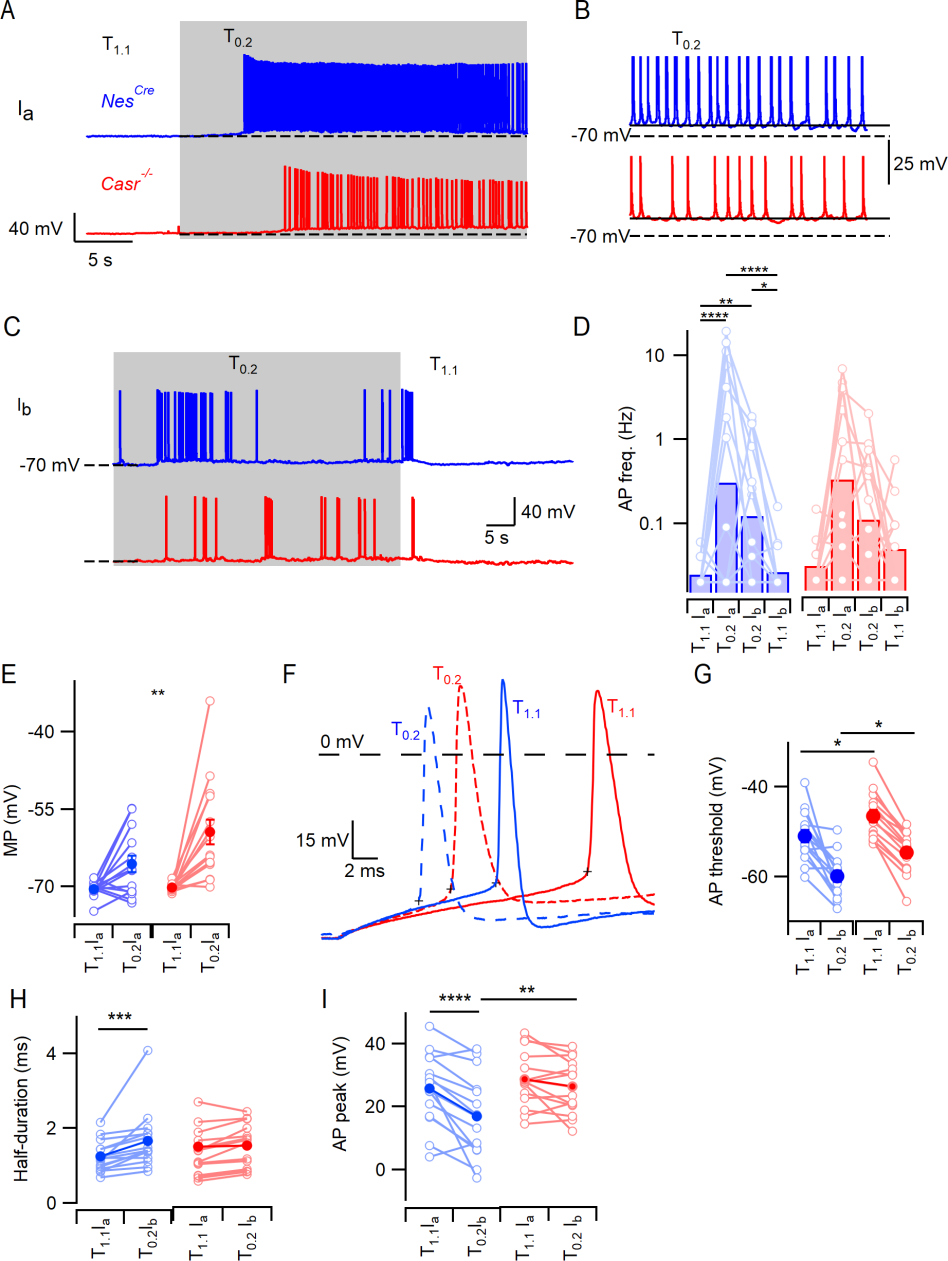
CaSR deletion does not affect divalent-dependent excitability at equivalent membrane potential. (**A**) Exemplary traces showing the divalent-dependent increase in neuronal excitability following the switch from T_1.1_ to T_0.2_ (change indicated by upper trace) in *Nes^Cre^* (blue) and *Casr^-/-^* (red) neurons when initial membrane potentials matched at −70 mV (broken line). (**B**) Expanded view of the final 5 s of traces in A illustrating sustained depolarization from following T_0.2_ application. (**C**) Exemplary traces showing the divalent-dependent decrease in neuronal excitability following the switch from T_0.2_ to T_1.1_ (change indicated by upper trace) in *Nes^Cre^* (blue) and *Casr^-/-^* (red) neurons when initial membrane potentials matched at −70 mV. Same recordings as A. (**D**) Histogram of average divalent-dependent changes in action potential frequency (Hz) in *Nes^Cre^* (blue) and C*asr^-/-^* (red) neurons when initial voltage is −70 mV in T_1.1_ (Ia) or T_0.2_ (Ib). Two-way RM ANOVA performed after logarithmic transformation indicates that reducing [Ca^2+^]_o_ increases the action potential frequency (F (3, 87)=17.97, p<0.0001) similarly in *Nes^Cre^* and *Casr^-/-^* neurons (F (1, 29)=0.2005, p=0.6577; Figure 2—source data 1). Post-hoc tests indicate significant differences between action potential frequency in T_1.1_ and T_0.2_ regardless of the holding current but not between action potential frequency recorded at different holding currents and the same solutions (Ia or Ib; Table 7). (**E**) Membrane potential depolarization following the switch to T_0.2_ from T_1.1_. Two-way RM ANOVA indicates that reducing [Ca^2+^]_o_ (F (1, 29)=29.22, p<0.0001) and CaSR deletion (F (1, 29)=4.874, p=0.0353) significantly depolarized the membrane potential but that there was no interaction (F (1, 29)=4.055, p=0.0534). Post-hoc testing indicate that membrane potentials were matched using current injection in T_1.1_ (-70.5 ± 0.4 mV and −70.2 ± 0.2 mV for *Nes^Cre^* and *Casr^-/-^* neurons respectively, p=0.985) but different in T_0.2_ (-65.6 ± 1.6 mV and –59.4 ± 2.4 mV, p=0.0083). (**F**) Exemplar action potentials elicited by current injection from −70 mV in a *Nes^Cre^* (blue) and a *Casr^-/-^* neuron (red) in solutions T_1.1_ (unbroken) and T_0.2_ (broken). Action potential threshold is indicated by +symbol for the first action potential elicited by current injection (50 to 200 pA). (**G**) Plot of average action potential threshold in T_1.1_ and T_0.2_ in *Nes^Cre^* and *Casr^-/-^* neurons, elicited as per panel F here and in subsequent panels. Two-way RM ANOVA indicates that reducing [Ca^2+^]_o_ hyperpolarized the action potential threshold (F (1, 25)=51.66, p<0.0001), whereas CaSR deletion had the opposite effect (F (1, 25)=10.52, p=0.0033). There was no interaction (Table 9A). Post-hoc tests indicate that the action potential thresholds in solutions T_1.1_ and T_0.2_ were depolarized similarly by CaSR deletion (5.3 ± 2.0 mV and 5.5 ± 2.0 mV, p=0.020 and 0.017) in *Nes^Cre^* and *Casr^-/-^* neurons, respectively. (**H**) Plot of average action potential half-duration in T_1.1_ and T_0.2_ in *Nes^Cre^* and *Casr^-/-^* neurons. Two-way RM ANOVA indicates that reducing [Ca^2+^]_o_ prolonged the action potential half-duration (F (1, 28)=19.73, p=0.0001). (**I**) Plot of average action potential peak in T_1.1_ and T_0.2_ in *Nes^Cre^* and *Casr^-/-^* neurons. The action potential peaks were higher in T_1.1_ and in Casr-/- neurons (Table 9C). Figure 2—source data 1.Action potential frequency in *Nes^Cre^* and *Casr^-/-^* neurons in T_1.1_ or T_0.2_ with standing currents I_a_ and I_b_.The action potential frequency is in log base 10 and each sub-column represents measurements from a single neuron.

Further mechanistic complexity was suggested by the effects of CaSR and [Ca^2+^]_o_ on RMP. This lead to a number of additional questions including: does the difference in RMP contribute to the difference in divalent-dependent excitability between *Nes^Cre^* and *Casr^-/-^* neurons, how do decreases in [Ca^2+^]_o_ depolarize *Casr^-/-^* neurons, and is this pathway present in *Nes^Cre^* neurons? To address the first of these questions, we compared the response of *Nes^Cre^* and *Casr^-/-^* neurons to changes in extracellular divalent concentrations after removing the confounding variation in RMP. After establishing a stable current-clamp recording in T_1.1_ we injected a standing current (I_a_) until the resting membrane potential was −70 mV. We then recorded for 50 s before switching the bath solution to T_0.2_. As before, there was a small depolarization followed by an increase in action potential frequency in *Nes^Cre^* neurons (Figure 2A,B). To test if this increase in excitability was fully attributable to divalent-dependent depolarization we adjusted the standing current (I_b_) until the membrane potential was −70 mV and then measured the action potential frequency (Figure 2C). In the exemplar, action potential firing was reduced by the hyperpolarization but remained higher in T_0.2_ at −70 mV than in T_1.1_ at −70 mV (Figure 2A–C) confirming CaSR-mediated depolarization was not acting alone to increase the excitability. The *Casr^-/-^* neurons responded similarly to T_0.2_ and hyperpolarization (Figure 2A–C) indicating the effect was not mediated by CaSR. We compared the average effects of T_1.1_ at −70 mV with I_a_, T_0.2_ with I_a_, and T_0.2_ at −70 mV with I_b_ on *Nes^Cre^* and *Casr^-/-^* genotypes (Figure 2D, Table 6) using a 2-way RM ANOVA. Extracellular divalent concentration and current injection substantially affected action potential frequency (F (3, 87)=17.97, p<0.0001). CaSR deletion did not impact the response to extracellular divalent concentration when *Nes^Cre^* and *Casr^-/-^* neuron recordings were started at a membrane potential of −70 mV l (F (1, 29)=0.2005, p=0.6577). Post-hoc testing showed that excitability was increased in T_0.2_ compared with T_1.1_ regardless which of the two holding currents were used (Figure 2D; Table 7). After injection of I_a_ to set the membrane potential to −70 mV, the switch from T_1.1_ to T_0.2_ still significantly depolarized the membrane potential (Figure 2E; Table 8, Two-way RM ANOVA, F (1, 29)=29.22, p<0.0001) as did CaSR deletion (F (1, 29)=4.874, p=0.0353). Post-hoc testing indicate that the membrane potential in T_0.2_ was more depolarized in the *Casr^-/-^* than in *Nes^Cre^* neurons (Figure 2B,E; −65.6 ± 1.6 mV vs −59.4 ± 2.4 mV, p=0.0083). Taken together, these experiments indicate CaSR-NALCN signaling was not contributing to the difference in divalent-dependent excitability between *Nes^Cre^* and *Casr^-/-^* neurons but that these differences may be due to genotype-dependent differences in RMP or intrinsic excitability.

**Table 6.** Action potential frequency.

ANOVA table	SS	DF	MS	F (DFn, DFd)	p Value
Interaction	0.4305	3	0.1435	F (3, 87)=0.3481	p=0.7906
[Ca^2+^]_o_ and I	22.23	3	7.410	F (3, 87)=17.97	p<0.0001
Genotype	0.1341	1	0.1341	F (1, 29)=0.2005	p=0.6577
Subjects (matching)	19.41	29	0.6692	F (29, 87)=1.623	p=0.0445
Residual	35.87	87	0.4123		

**Table 7.** Action potential frequency.

Sidak's multiple comparisons test	Mean diff.	95% CI of diff.	Significant?	Summary	Adjusted p value
T_1.1_ Ia vs. T_0.2_ Ia	−1.059	−1.498 to −0.6200	Yes	****	<0.0001
T_1.1_ Ia vs. T_0.2_ Ib	−0.6203	−1.059 to −0.1813	Yes	**	0.0016
T_1.1_ Ia vs. T_1.1_ Ib	−0.1163	−0.5554 to 0.3227	No	ns	0.9797
T_0.2_ Ia vs. T_0.2_ Ib	0.4387	−0.0003709 to 0.8778	No	ns	0.0503
T_0.2_ Ia vs. T_1.1_ Ib	0.9427	0.5037 to 1.382	Yes	****	<0.0001
T_0.2_ Ib vs. T_1.1_ Ib	0.5040	0.06496 to 0.9431	Yes	*	0.0160

**Table 8.** Membrane potential with Ia.

ANOVA table	SS	DF	MS	F (DFn, DFd)	p Value
Interaction	131.7	1	131.7	F (1, 29)=4.055	p=0.0534
[Ca^2+^]_o_	949.2	1	949.2	F (1, 29)=29.22	p<0.0001
Genotype	162.7	1	162.7	F (1, 29)=4.874	p=0.0353
Subjects (matching)	968.0	29	33.38	F (29, 29)=1.028	p=0.4711
Residual	942.0	29	32.48		

### Voltage-gated sodium channels contribute to divalent-dependent excitability

Reversal of the divalent-dependent depolarization did not completely block the increased excitability associated with the switch to T_0.2_ (Figures 1E, F and 2D) indicating another mechanism other than NALCN was responsible. We tested if voltage-gated channels were contributing by to divalent-dependent excitability by examining action potential threshold in neurons held at a membrane potential of −70 mV. Action potential threshold was hyperpolarized by 8 mV on average following the change from T_1.1_ to T_0.2_ in *Nes^Cre^* and *Casr^-/-^* neurons (Figure 2F,G, Table 9A; F (1, 25)=51.66, p<0.0001). Furthermore, the action potential threshold was relatively depolarized in the *Casr^-/-^* neurons in T_1.1_ and T_0.2_ (5.3 ± 2.0 mV (p=0.020) and 5.5 ± 2.0 mV (p=0.017) respectively), indicating *Nes^Cre^* neurons possessed increased excitability and increased sensitivity to decreases in external divalent concentration (Figure 2F,G). The action potential half-width recorded under the same conditions, was also sensitive to the reduction of divalent concentration but unaffected by CaSR deletion (Figure 2H,I Table 9B). ANOVA indicated that the switch to T_0.2_ from T_1.1_ broadened action potential half-width (F (1,28)=19.7, p=0.0001). The genotype and [Ca^2+^]_o_ interacted to both affect action potential peak voltage (Figure 2I, Table 9C; F (1, 28)=6.76, p=0.015) with the peak potential being reduced by T_0.2_ in the *Nes^Cre^* (p<0.0001) but not *Casr^-/-^* neurons (p=0.34).

**Table 9.** Action potential threshold recorded at −70 mV.

ANOVA table	SS	DF	MS	F (DFn, DFd)	p Value
Interaction	0.06658	1	0.06658	F (1, 25)=0.004070	p=0.9496
[Ca^2+^]_o_	845.1	1	845.1	F (1, 25)=51.66	p<0.0001
Genotype	391.0	1	391.0	F (1, 25)=10.52	p=0.0033
Subjects (matching)	929.5	25	37.18	F (25, 25)=2.273	p=0.0225
Residual	408.9	25	16.36		
(B) Action potential threshold recorded at −70 mV					
Interaction	2.008e-07	1	2.008e-07	F (1, 28)=2.800	p=0.1054
[Ca^2+^]_o_	1.415e-06	1	1.415e-06	F (1, 28)=19.73	p=0.0001
Genotype	3.050e-07	1	3.050e-07	F (1, 28)=0.4545	p=0.5057
Subjects (matching)	1.879e-05	28	6.710e-07	F (28, 28)=9.358	p<0.0001
Residual	2.008e-06	28	7.170e-08		
(C) Action potential threshold recorded at −70 mV					
Interaction	0.0001602	1	0.0001602	F (1, 28)=6.758	p=0.0147
[Ca^2+^]_o_	0.0004821	1	0.0004821	F (1, 28)=20.34	p=0.0001
Genotype	0.001193	1	0.001193	F (1, 28)=5.891	p=0.0219
Subjects (matching)	0.005669	28	0.0002025	F (28, 28)=8.541	p<0.0001
Residual	0.0006637	28	2.370e-005		

**Figure 3.**
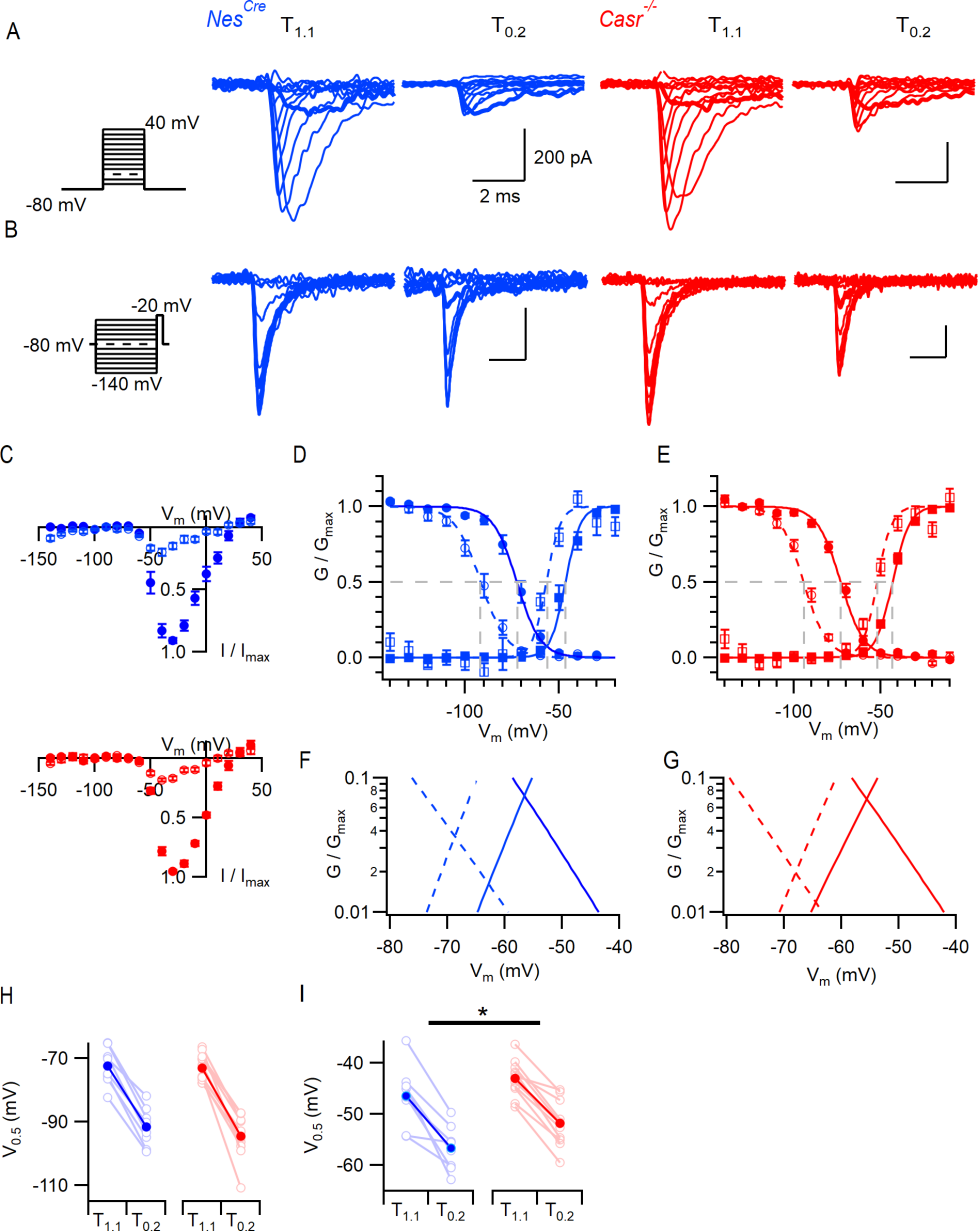
CaSR deletion and external divalent concentration affect VGSC current gating. (**A**) Exemplary traces showing VGSC currents activated by voltage steps from −80 in 10 mV increments (left), in nucleated patches isolated from *Nes^Cre^* (blue) and *Casr^-/-^* (red) neurons in solutions T_1.1_ and T_0.2_. The VGSC currents elicited by 10 ms depolarizations to −50 mV (bold) were greater following the switch to T_0.2_. (**B**) Exemplary traces showing VGSC currents activated by voltage steps to −20 mV following a 100 ms conditioning step (left), in the same patches as (A) using solutions T_1.1_ and T_0.2_. The VGSC currents elicited following conditioning steps to −80 mV (bold) were smaller following the switch to T_0.2_. (**C**) Current-voltage plots of average normalized VGSC currents in nucleated patches from *Nes^Cre^* (n = 8) and *Casr^-/-^* (n = 11) neurons in T_1.1_ (filled circles) and T_0.2_ (open circles). Currents were normalized using the maximum VGSC current in each recording. (**D**) Plot of average normalized conductance versus voltage in patches from *Nes^Cre^* neurons for activation (square, n = 8) and inactivation (circle, n = 8) in solutions T_1.1_ (filled) and T_0.2_ (open). Boltzmannn curves are drawn using average values from individual fits and gray broken lines indicate V_0.5_ values for each condition. (**E**) Plot of average normalized conductance versus voltage in patches from *Casr^-/-^* neurons for activation (square, n = 11) and inactivation (circle, n = 12) in solutions T_1.1_ (filled) and T_0.2_ (open). Boltzmannn curves are drawn using average values from individual fits and gray broken lines indicate V_0.5_ values for each condition. Inset shows plot expanded to emphasize voltage dependence of the window currents. (**F** and **G**) represent the plots of D and E expanded to emphasize the voltage dependence of the window currents. (**H**) Histogram showing V_0.5_ for VGSC inactivation in T_1.1_ and T_0.2_ in patches from *Nes^Cre^* and *Casr^-/-^* neurons. (**I**) Histogram showing V_0.5_ for VGSC activation in T_1.1_ and T_0.2_ in patches from *Nes^Cre^* and *Casr^-/-^* neurons.

We examined the properties of VGSCs and voltage-gated potassium channels (VGPCs) to determine the reason for the altered action potential threshold. VGSCs were isolated in neocortical neurons and the current-voltage characteristics examined. Families of VGSC currents were activated in neurons after 2–4 weeks in culture. Maximum VGSC currents were elicited at −30 mV and averaged −8.0 ± 0.8 nA (n = 7) and −8.8 ± 2.8 nA (n = 6) in *Nes^Cre^* and *Casr^-/-^* neurons, respectively. The current-voltage curve shifted in a hyperpolarizing direction with the switch from T_1.1_ to T_0.2_ but extensive neuronal processes limited the quality of the voltage-clamp and prevented useful analysis. We examined VGSC gating in nucleated outside-out patches (Sather et al., 1992; Almog et al., 2018) to ensure better voltage control. VGSC currents were elicited by voltage steps from −80 mV (10 mV increments to 40 mV). In T_0.2_, the VGSC inactivation (see below) resulted in smaller currents that were more sensitive to depolarization (bold traces elicited by steps to −50 mV, Figure 3A) as previously observed (Frankenhaeuser and Hodgkin, 1957; Campbell and Hille, 1976; Armstrong and Cota, 1991). Divalent sensitivity was confirmed in the normalized current-voltage plot for both *Nes^Cre^* (blue, n = 8) and *Casr^-/-^* (red, n = 11) neurons (Figure 3A,C). VGSC current inactivation was studied using a test pulse to −20 mV, each of which was preceded by a conditioning step (100 ms) to between −140 mV and −20 mV. In T_1.1_ we observed less inactivation than in T_0.2_ (Figure 3B, bold traces show currents elicited following prepulse to −80 mV). We compared the effects of [Ca^2+^]_o_ and CaSR deletion on VGSC current inactivation using plots of normalized conductance and measuring the half maximal voltage (V_0.5_; circles, Figure 3D,E). The reduction in divalent concentration left-shifted V_0.5_ (F (1, 18)=56, p<0.0001, 2-way RM ANOVA, Table 10) but CaSR deletion did not (F (1, 18)=0.563, p=0.463). The switch from T_1.1_ to T_0.2_ shifted V_0.5_ by −20 and −21 mV in *Nes^Cre^* and *Casr^-/-^*, respectively (−72 ± 2 to −92 ± 2 mV and −73 ± 1 to −94 ± 2 mV).

**Table 10.** Voltage-gated sodium channel current V_0.5_ for inactivation.

ANOVA table	SS	DF	MS	F (DFn, DFd)	p Value
Interaction	12.00	1	12.00	F (1, 18)=0.7743	p=0.3905
[Ca^2+^]_o_	3973	1	3973	F (1, 18)=256.2	p<0.0001
Genotype	27.49	1	27.49	F (1, 18)=0.5632	p=0.4627
Subjects (matching)	878.5	18	48.81	F (18, 18)=3.148	p=0.0097
Residual	279.1	18	15.50		

We also tested how the VGSC activation was affected by CaSR and [Ca^2+^]_o_. The peak inward VGSC currents (Figure 3B,D) were divided by the driving voltage and then plotted as conductance-voltage plots. The normalized conductance plots (squares, Figure 3E,F) indicate that the switch from T_1.1_ to T_0.2_ significantly facilitated VGSC activation consistent with VGSCs in other excitable cells (V_0.5_ was hyperpolarized by 10 mV; F (1, 17)=98, p<0.0001; two-way RM ANOVA, Table 11; Hille, 2001). Switching from T_1.1_ to T_0.2_ shifted V_0.5_ by −11 mV and −9 mV in *Nes^Cre^* and *Casr^-/-^* neurons respectively (−46 ± 2 to −57 ± 2 mV and −43 ± 1 to −52 ± 1 mV). The unexpected shift in V_0.5_ for VGSC activation in Casr^-/-^ neurons will reduce the likelihood of VGSC activation (F (1, 17)=4.8, p=0.04) in these cells (Figure 3I). Overlap of the inactivation and activation conductance plots represents the voltage range over which persistent VGSC currents, or window currents, are likely to occur (Chadda et al., 2017). Divalent reduction hyperpolarized this region of overlap toward the RMP (Figure 3F,G insets) increasing the likelihood that persistent VGSC currents were activated at resting membrane potential and therefore contributing to divalent-dependent excitability. The depolarization of VGSC activation gating that resulted from CaSR deletion (Figure 3I), shifted the area of conductance curve overlap for T_0.2_ in a depolarizing direction (Figure 3G). This effect would reduce the fraction of VGSCs available for activation by T_0.2_ at the more hyperpolarized RMPs and explain the reduced the likelihood of spontaneous action potential generation in Casr^-/-^ neurons (Figure 1).

**Table 11.** Voltage-gated sodium channel current V_0.5_ for activation.

ANOVA table	SS	DF	MS	F (DFn, DFd)	p Value
Interaction	4.814	1	4.814	F (1, 17)=0.5668	p=0.4618
[Ca^2+^]_o_	834.5	1	834.5	F (1, 17)=98.24	p<0.0001
Genotype	157.6	1	157.6	F (1, 17)=4.813	p=0.0424
Subjects (matching)	556.7	17	32.75	F (17, 17)=3.855	p=0.0040
Residual	144.4	17	8.494		

**Figure 4.**
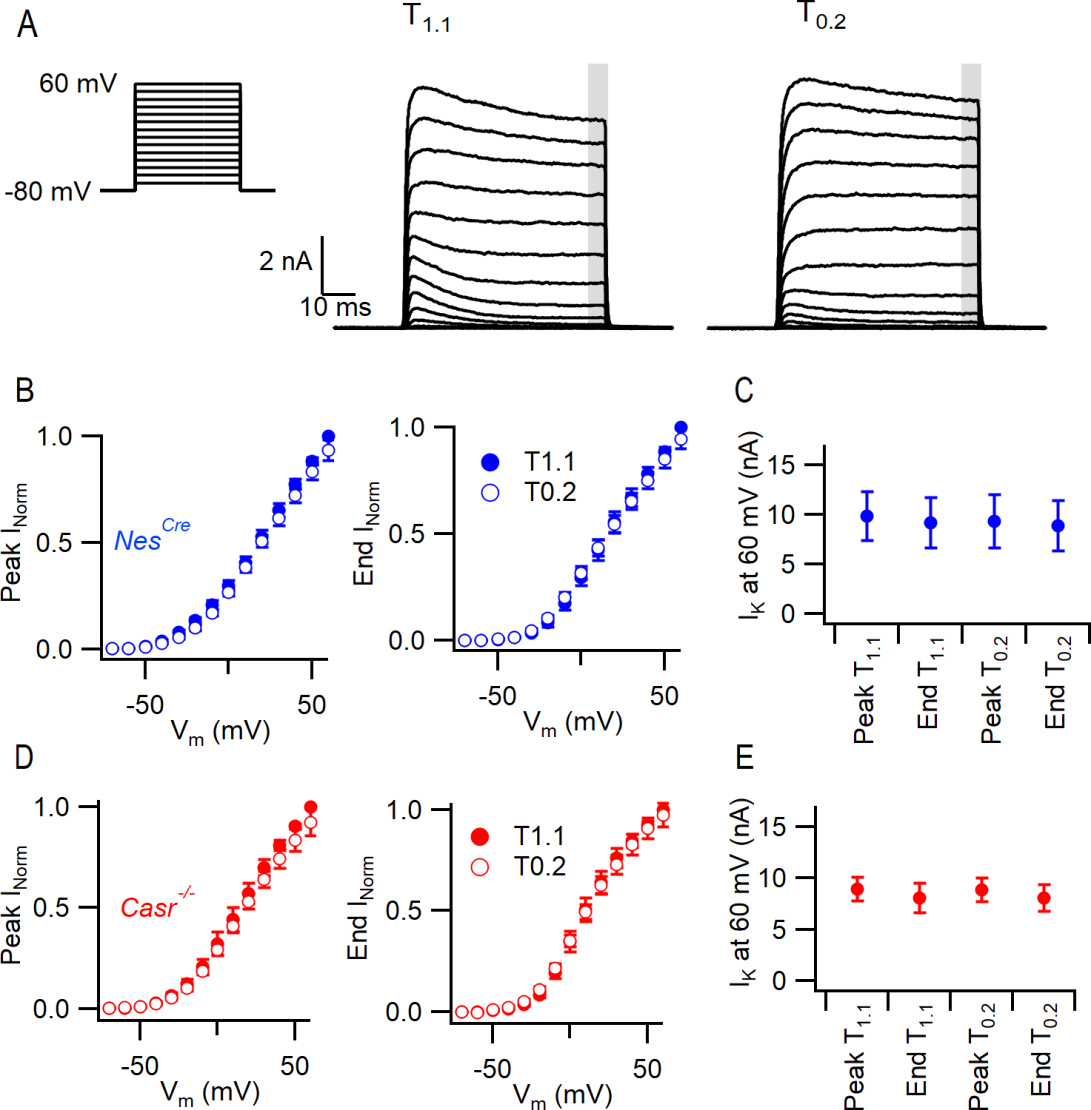
CaSR deletion and external divalent concentration do not significantly affect VGPC current gating. (**A**) Exemplary traces showing VGPC currents activated by voltage steps from −80 in 10 mV increments (left), in a *Nes^Cre^* neuron in solutions T_1.1_ and T_0.2_. The outward currents elicited by the 50 ms voltage step were measured at peak and at the end of the step (average of last 5 ms indicated by gray bar). (**B**) Current voltage-plot of average normalized VGPC currents (n = 10) in *Nes^Cre^* neurons in T_1.1_ (filled circles) and T_0.2_ (open circles) at peak or end of step. Currents were normalized using the maximum outward current in each condition here and below. (**C**) Peak and end outward currents at 60 mV elicited in same neurons as B. Two-way RM ANOVA indicates that peak and outward currents were not different in T_1.1_ or T_0.2_ ((3, 57)=1.347), p=0.2683 nor were they affected by CaSR deletion (data from E, (1, 19)=1.231, p=0.2811). (**D**) Current voltage-plot of average normalized VGPC currents (n = 11) in *Casr^-/-^* neurons in T_1.1_ (filled circles) and T_0.2_ (open circles) at peak or end of step. (**E**) Peak and end outward currents at 60 mV elicited in same neurons as D.

VGPC currents were isolated and recorded in *Nes^Cre^* and *Casr^-/-^* neurons in T_1.1_ and T_0.2_ solutions after blocking contaminating currents. Currents were elicited by a series of 60 ms steps from −70 mV to 60 mV in 10 mV increments (Figure 4). The VGPC current amplitudes were measured at the peak and at the end of the depolarizing step (normalized to the value at 60 mV in T_1.1_). Neither the peak nor end current were affected by reduction of the external divalent concentration or by deletion of CaSR (Figure 4) over the range of voltages. The currents activated at 60 mV were similarly unaffected (Figure 4, Two-way RM ANOVA [(3, 57)=1.347, p=0.2683 and (1, 19)=1.231, p=0.2811, Table 12]). These data indicate that VGPCs are not involved in divalent-dependent excitability in neocortical neurons.

**Table 12.** Voltage-gated potassium channel currents at 60 mV.

ANOVA table	SS	DF	MS	F (DFn, DFd)	p Value
Interaction	1.226e-018	3	4.086e-019	F (3, 57)=0.2271	p=0.8772
[Ca^2+^]_o_ and time	7.270e-018	3	2.423e-018	F (3, 57)=1.347	p=0.2683
Genotype	6.054e-017	1	6.054e-017	F (1, 19)=1.231	p=0.2811
Subjects (matching)	9.345e-016	19	4.919e-017	F (19, 57)=27.33	p<0.0001
Residual	1.026e-016	57	1.800e-018		

### VGSCs are the dominant contributor to divalent-dependent currents

**Figure 5.**
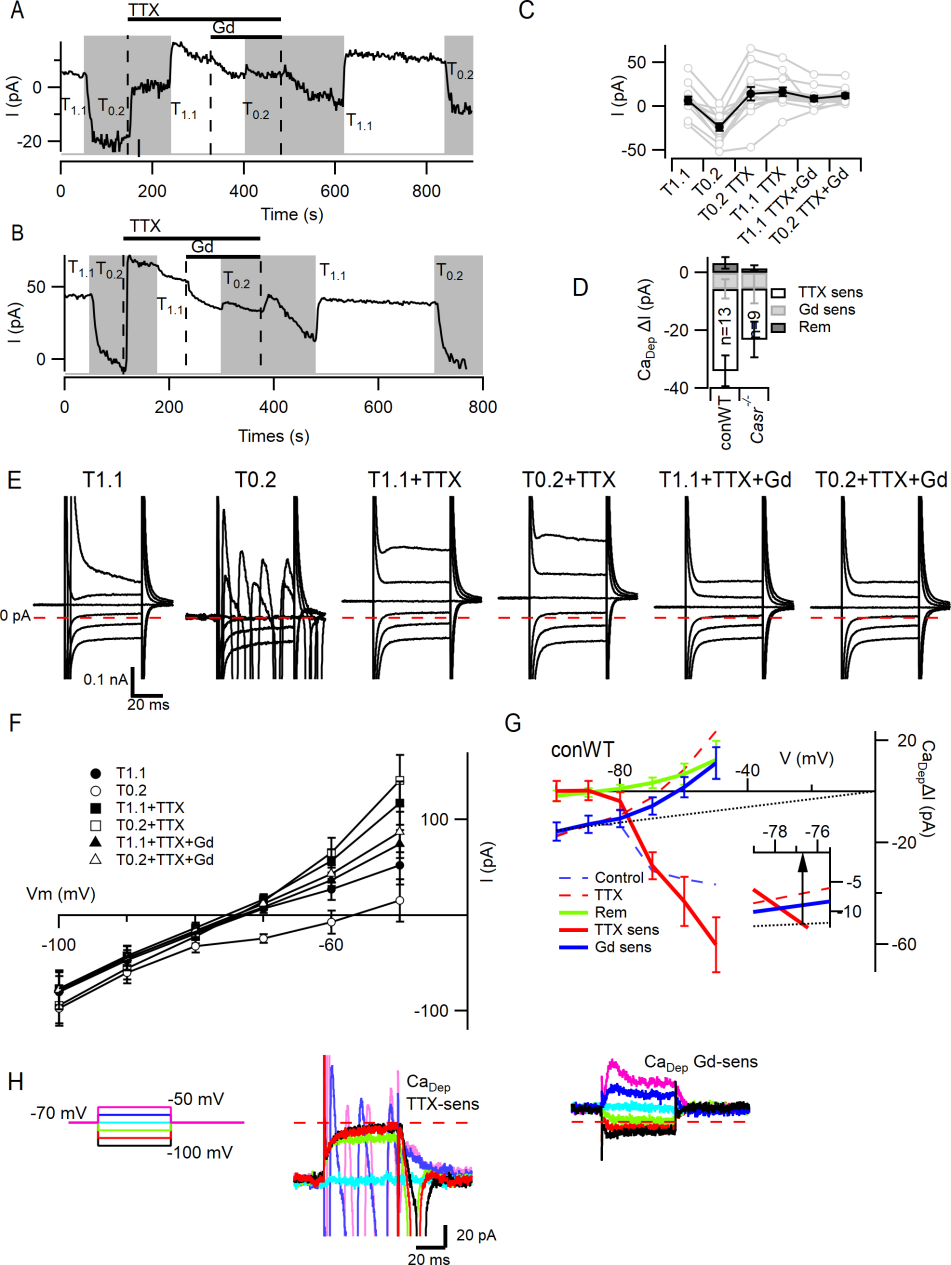
VGSC current activation by decreased external divalent concentration. (**A,B**) Plots illustrating the responses of the basal currents in two WT neurons during application of T_1.1_ and T_0.2_ before and during TTX or TTX and Gd^3+^. Average basal currents were measured over 50 ms every 2 s with T_1.1_ and T_0.2_ application indicated by vertical shading (gray represents T_0.2_) and blockers application by horizontal bars and broken vertical lines. (**C**) Plot of average basal current measurements (filled circles) and individual neurons (open circles) in each solution condition in conWT (n = 13) neurons. Each basal current represents the average value recorded during last 20 s of the specific solution application. (**D**) Average [Ca^2+^]_o_ dependent basal currents sensitive to TTX and Gd^3+^ calculated by subtraction of data in C and the remaining [Ca^2+^]_o_ dependent current after application of both blockers for conWT neurons. (**E**) Exemplar traces of currents elicited by 50 ms voltage steps between −100 and −50 mV during application of solutions described in C. (**F**) Plots of the average currents over the last 5 ms of each voltage step in all six solutions for conWT (n = 13). (**G**) Plots of the average [Ca^2+^]_o_ dependent currents derived by subtraction of conWT data (**F**) resolved as total or control (broken blue), in the presence of TTX (broken red), and in the presence of TTX and Gd^3+^ (remainder green). The TTX-sensitive (solid red), Gd^3+^-sensitive (solid blue) and NALCN (dotted line) component currents were obtained by further subtraction. Inset shows expanded view at intercept of TTX-sensitive and NALCN components. (**H**) Exemplars of the TTX- and Gd^3+^-sensitive [Ca^2+^]_o_-dependent currents. Broken red line represents zero current line.

To compare the contributions of VGSCs and NALCN to the divalent-dependent depolarization seen in neocortical neurons (Figure 2), we measured the size of the currents elicited at −70 mV in neurons following the switch from T_1.1_ to T_0.2_. We used conWT neurons to avoid potential confounding Cre-dependent effects (Qiu et al., 2011). Since NALCN is resistant to the VGSC blocker tetrodotoxin (TTX) (Lu et al., 2007; Swayne et al., 2009) but Gd^3+^ (10 µM) inhibits NALCN and VGSCs (Elinder and Arhem, 1994; Li and Baumgarten, 2001; Lu et al., 2009), we were able to pharmacologically separate the contributions of VGSCs and NALCN to the basal current following the switch from T_1.1_ to T_0.2_ (-31 ± 3 pA, n = 13; Figure 5A–C). Addition of a saturating concentration of TTX (1 µM) in T_0.2_ inhibited a persistent inward current within a few seconds in all but one of the recordings (Figure 5A–C), consistent with VGSCs contributing to the inward current elicited by T_0.2_. Switching to T_1.1_ plus TTX produced minimal change in the basal current on average (Figure 5C). However, in some neurons, T_1.1_ elicited an outward current (Figure 5A,C), whereas in others there was an inward current (Figure 5B,C) indicating the presence of two types of TTX resistant divalent-sensitive pathways. Presumably, NALCN was contributing to the divalent-dependent TTX-resistant effect observed in Figure 5A. Co-application of Gd^3+^ (10 µM) following block of VGSCs with TTX, resulted in a small inward deflection of the average basal current in solution T_1.1_ and largely inhibited sensitivity to concomitant decreases in [Ca^2+^]_o_ (Figure 5A–C). The reduced sensitivity of neurons to the reduction of [Ca^2+^]_o_ in the presence of TTX, suggests that VGSCs are a major contributor to the depolarizing current elicited by low [Ca^2+^]_o._ Using serial subtraction of the basal currents (Figure 5C), we compared the size of the TTX-sensitive (−28.2 ± 5.3 pA), Gd^3+^-sensitive (−5.7 ± 3.4 pA) and remaining (3.4 ± 2.0 pA) divalent-dependent currents (Figure 5D; RM-ANOVA, F (1.495, 17.94)=13.30, p=0.0007, Table 13). Multiple comparison testing indicated that the TTX-sensitive divalent-dependent current was greater than the Gd^3+^-sensitive (p=0.039) and remaining divalent-dependent currents (p=0.0009; Table 14). Similar differences in the relative sizes of the TTX-, Gd^3+^-, and remainder divalent-dependent basal current currents were also observed in Casr^-/-^neurons (Figure 5D). While there were rare neurons in which there was a larger Gd^3+^-sensitive current (Figure 5C) the reduced sensitivity of neurons to the reduction of [Ca^2+^]_o_ in the presence of TTX, confirms that VGSCs are the major contributor to the depolarizing current elicited by low [Ca^2+^]_o_.

**Figure 5—figure supplement 1.**
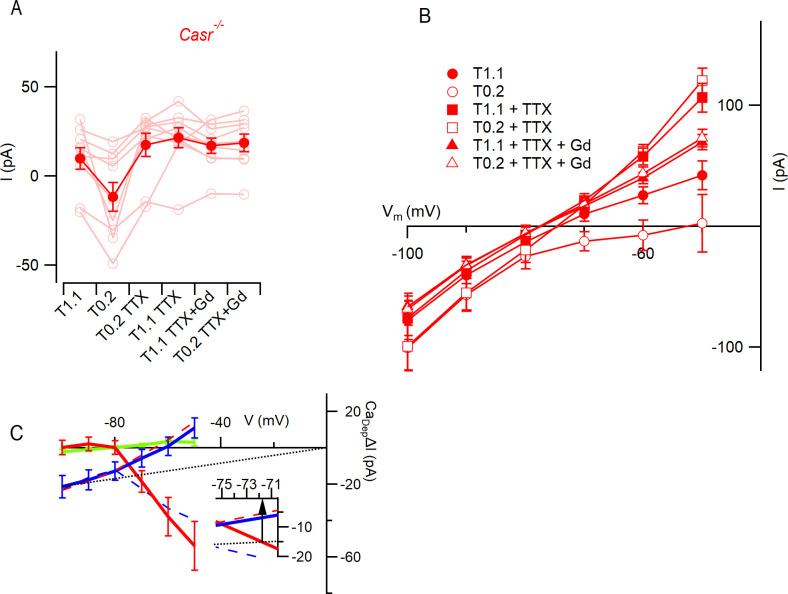
VGSC current activation by decreased external divalent concentrationin *Casr^-/-^* neurons. (**A**) Plot of average basal current measurements (filled circles) and individual neurons (open circles) in each solution condition in *Casr^-/-^* (n = 9) neurons. Each basal current represents the average value recorded during last 20 s of the specific solution application. (**B**) Plots of average currents versus voltage for *Casr^-/-^* (n = 9) neurons as per Figure 5F (**C**) Plots of the average [Ca^2+^]_o_-dependent currents derived by subtraction of *Casr^-/-^* data as per Figure 5G.

**Table 13.** divalent-dependent basal currents at −70 mV.

ANOVA table	SS	DF	MS	F (DFn, DFd)	p Value
Treatment	6.871e-021	2	3.435e-021	F (1.495, 17.94)=13.30	p=0.0007
Individual (between rows)	5.669e-022	12	4.725e-023	F (12, 24)=0.1828	p=0.9981
Residual (random)	6.201e-021	24	2.584e-022		
Total	1.364e-020	38			

**Table 14.** Post hoc testing of divalent-dependent basal currents at −70 mV.

Sidak's multiple comparisons test	Mean diff.	95% CI of diff.	Significant?	Summary	Adjusted p value
TTX sens vs. Gd^3+^ sens	−2.247e-011	−4.391e-011 to −1.033e-012	Yes	*	0.0392
TTX sens vs. Rem	−3.158e-011	−4.899e-011 to −1.418e-011	Yes	***	0.0009
Gd^3+^ sens vs. Rem	−9.111e-012	−2.146e-011 to 3.240e-012	No	ns	0.1789

In a fraction of the neurons, an inward deflection of the basal current occurred when external divalent concentration was increased in the presence of TTX (Figure 5B,C) which contrasted with the outward current expected from NALCN deactivation (Figure 5A). We examined the voltage-dependence of the contributions of VGSCs, NALCN, and this second divalent-dependent TTX-resistant current to better characterize divalent-dependent excitability. We used 50 ms voltage steps between −100 and −50 mV and averaged the current over the last 5 ms of the step. Three additional major effects are illustrated by the exemplar current traces (Figure 5E). First, in the absence of blockers, the switch from T_1.1_ to T_0.2_ substantially increased the number of large, rapidly inactivating inward currents even at −70 mV following hyperpolarizing steps. Second, in TTX, low [Ca^2+^]_o_ increased the linear inward and rectifying outward currents. Third, in the presence of TTX and Gd^3+^ changing between T_1.1_ and T_0.2_ had little effect suggesting Gd^3+^ is blocking both NALCN and the second divalent-dependent TTX-resistant current. These observations were confirmed in the average current-voltage plots (Figure 5F) where it is clear that at −80 to −100 mV the major divalent-dependent currents are inward and resistant to TTX and sensitive to Gd^3+^, whereas at −70 to −50 mV the largest divalent-dependent currents are TTX-sensitive. The divalent-dependent effects were calculated by subtracting the currents recorded in T_1.1_ from those in T_0.2_ under control conditions (Figure 5G, broken red), in the presence of TTX (broken blue) and TTX plus Gd^3+^ (solid green). The TTX-sensitive (solid red) and Gd^3+^-sensitive (solid blue) divalent-dependent currents were obtained by additional subtraction (broken red minus broken blue and broken blue minus green). The average divalent-dependent current carried by VGSCs only became evident once the neurons were depolarized above −80 mV (Figure 5G). The time course of deactivation of the persistent divalent-dependent VGSC currents was observed following hyperpolarization from −70 mV (Figure 5H, middle). At more negative potentials, the Gd^3+^-sensitive current accounted for all the divalent-dependent current and traces showed an ohmic voltage dependence (Figure 5G). However, the Gd^3+^-sensitive current reversed at −60 mV and outward currents were elicited by steps to −60 and −50 mV that exhibited a voltage-dependent activation and inactivation (Figure 5H, right-hand). This is consistent with the Gd^3+^-sensitive current consisting of the sum of NALCN and an outward voltage-dependent current. Assuming conservatively that all of the Gd^3+^-sensitive current at −100 mV could be attributed to NALCN and employing the channel’s linear voltage-dependence and zero mV reversal potential (Lu et al., 2007; Lu et al., 2010), then the amplitude of NALCN currents could be estimated over the voltage range −100 to 0 mV (broken black line, Figure 5G). By interpolation (Figure 5G, inset), the contribution of NALCN and VGSCs to divalent-dependent currents were equal at −77 mV with the contribution from VGSCs increasing with depolarization. A similar analysis of divalent-dependent currents in *Casr^-/-^* neurons indicated that the contribution of VGSCs was greater than that of NALCN once membrane potentials were depolarized beyond −72 mV (Figure 5—figure supplement 1). These data indicate that divalent-dependent currents around the resting membrane that contribute to divalent-dependent excitability are mainly attributable to VGSCs.

### Changes in resting potential resulting from lowered divalents are mediated mainly by VGSCs

**Figure 6.**
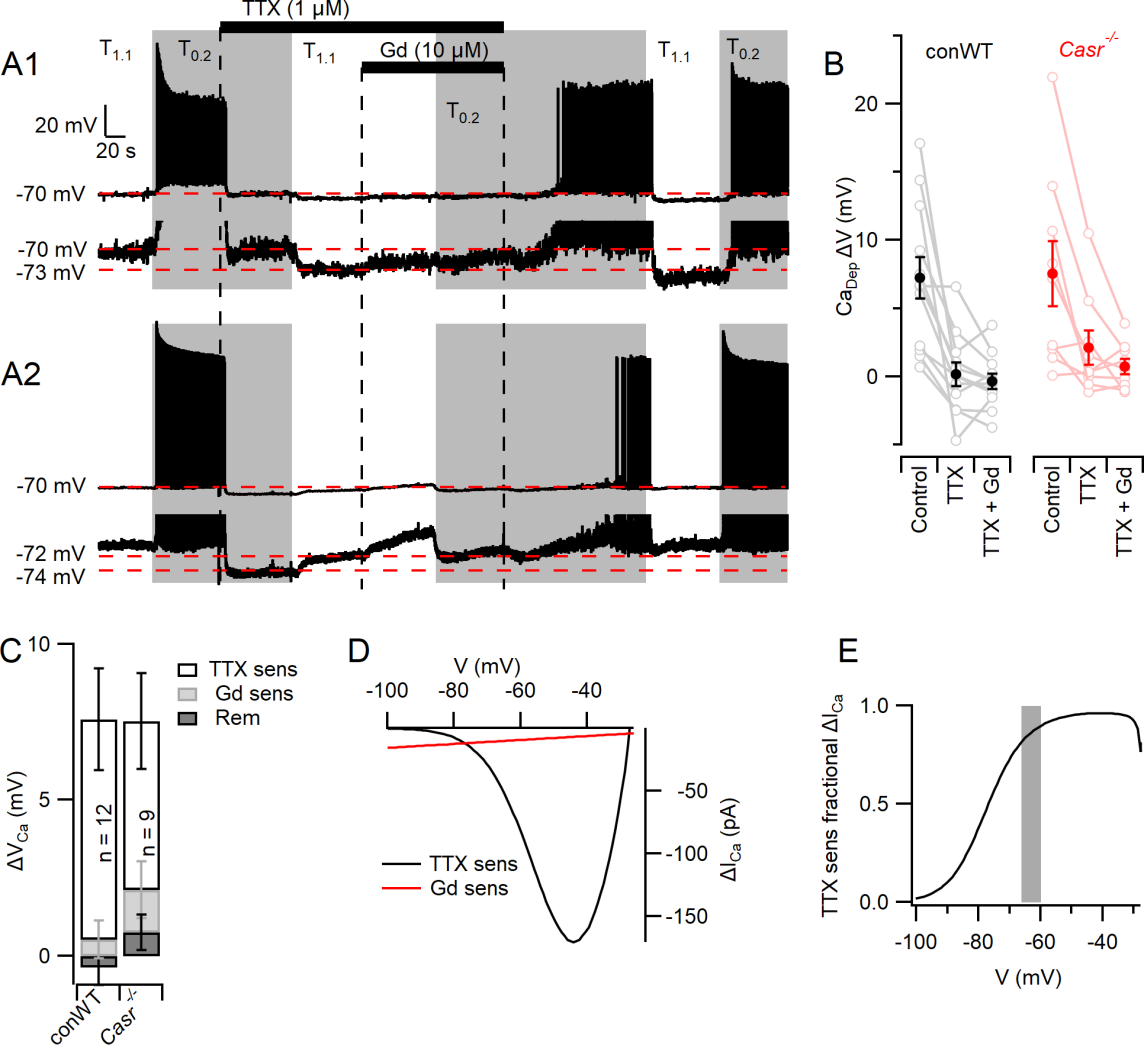
Divalent-dependent depolarization is almost entirely mediated via VGSCs. (**A**) The response of the membrane potential in two WT neurons during application of T_1.1_ and T_0.2_ before and during TTX or TTX and Gd^3+^. T_1.1_ and T_0.2_ application is indicated by vertical shading (gray represents T_0.2_) and blocker applications by horizontal bars and broken vertical lines. The broken red line indicates −70 mV. Voltage-expanded view of the trace illustrates that in the presence of TTX, hyperpolarization (A1) and depolarization (A2) may occur following the switch to T_1.1_. Membrane potential values highlighted by broken red lines. (**B**) Plot of average (filled circles) and individual (open circles) Ca^2+^-dependent voltage changes (filled circles) following the switch from T_1.1_ to T_0.2_ (by subtraction of average between-spike membrane potential over the last 10 s of each solution application). Each solution applied to conWT (n = 12) and *Casr^-/-^* (n = 9) neurons. (**C**) Average [Ca^2+^]_o_ dependent voltage changes sensitive to TTX and Gd^3+^ calculated by subtraction of data in B, and the remaining [Ca^2+^]_o_-dependent voltage after application of both blockers (Figure 6—source data 1). (**D**) Estimates of the average relative size of the external divalent concentration-dependent NALCN and VGSC currents in neocortical neurons between −100 and −30 mV. NALCN values from Figure 5G. The external divalent concentration-dependent VGSC currents were estimated as follows: the products of the VGSC activation and inactivation conductance plots were calculated for T_1.1_ and T_0.2_ using the average Boltzmann curves in Figure 3. These were converted to currents (I = driving voltage x conductance), and scaled to match the average TTX-sensitive current at −70 mV. The current generated in T_0.2_ minus that generated in T_1.1_ (ΔI_Ca_) was plotted against membrane voltage. (**E**) Plot of the average divalent-dependent depolarizing current carried by VGSC derived from D. The change in average resting membrane potential recorded in Figure 1 is indicated by the gray bar. Figure 6—source data 1.Depolarization elicited by switch from T_1.1_ to T_0.2_ that was sensitive to TTX, Gd^3+^, or resistant to both blockers in conventional WT and *Casr^-/-^* neurons.Depolarization units are volts and each sub-column represents measurements from a single neuron.

The complex architecture of neocortical neurons restricted our ability to clamp the membrane potential following the activation of large, rapid VGSC currents. Thus, we re-examined the contribution of VGSCs and NALCN to the depolarizations that mediate divalent-dependent excitability in current clamp recordings from conWT neurons. Consistent with earlier experiments (Figure 2), switching from T_1.1_ to T_0.2_ depolarized the membrane potential from −70 mV by 7.2 ± 1.5 mV (n = 12) and increased spontaneous action potential firing in pharmacologically isolated neurons (Figure 6A,B). We used TTX and Gd^3+^ to measure the contributions of VGSCs and NALCN respectively to these divalent-dependent depolarizations. TTX blocked action potential generation, as expected, but also hyperpolarized the membrane potential indicating that VGSCs were open in T_0.2_ (Figure 6A1) and T_1.1_ (Figure 6A2). The switch from T_0.2_ to T_1.1_ in TTX resulted in a hyperpolarization, consistent with divalent-dependent NALCN closure, in some neurons (Figure 6A 1 lower trace and B). Other neurons depolarized with the switch to T_1.1_ (Figure 6A 2 lower trace and B) consistent with a divalent-dependent outward current similar to that observed in Figure 5B,C. On average the divalent-dependent depolarization was almost entirely prevented by TTX or TTX and Gd^3+^ co-application (Figure 6B). The amplitude of the divalent-dependent depolarizations in conWT neurons changed with blocker type (1-way RM ANOVA, F (1.219, 13.41)=12.83, p=0.0022, Table 15). The TTX-sensitive component was greater than the Gd^3+^-sensitive and the blocker-resistant component (p=0.022 and 0.0028 respectively, Table 16). On average VGSCs accounted for 93% of the depolarization that triggers divalent-dependent excitability in WT neurons starting at −70 mV (Figure 6C) and we observed a similar pattern in Casr^-/-^ neurons (Figure 6C).

**Table 15.** divalent-dependent depolarization.

ANOVA table	SS	DF	MS	F (DFn, DFd)	p Value
Treatment	0.0003944	2	0.0001972	F (1.219, 13.41)=12.83	p=0.0022
Individual (between rows)	0.0001037	11	9.423e-006	F (11, 22)=0.6132	p=0.7982
Residual (random)	0.0003381	22	1.537e-005		
Total	0.0008361	35			

**Table 16.** Post hoc testing of blocker sensitive fractions of the divalent-dependent depolarization.

Sidak's multiple comparisons test	Mean diff.	95% CI of diff.	Significant?	Summary	Adjusted p value
TTX sens vs. Gd^3+^ sens	0.006528	0.001005 to 0.01205	Yes	*	0.0215
TTX sens vs. Rem	0.007428	0.002879 to 0.01198	Yes	**	0.0028
Gd^3+^ sens vs. Rem	0.0009	−0.001301 to 0.003101	No	ns	0.5311

Next we estimated the average relative contributions of the divalent-dependent NALCN and VGSC currents over a wider voltage range. Ohmic divalent-dependent NALCN currents were extrapolated from −100 mV, where contaminating currents appear minimal (Figure 5G) and compared with the divalent-dependent VGSC currents predicted from scaled conductance plots (Figure 3D). The VGSC currents were the major contributor to divalent-dependent currents over the −77 to −30 mV voltage range (Figure 6D,E). These findings indicate that VGSCs are the predominant contributor to the depolarizations that lead to action potential generation at lower external divalent concentrations (Figure 6E, gray bar).

## Discussion

Extracellular calcium concentration regulates both synaptic transmission and intrinsic neuronal excitability, thereby strongly affecting the probability of action potential generation. Consequently, physiological and pathological changes in [Ca^2+^]_o_ will impact neuronal computation in a complex manner. We have investigated the mechanisms underlying divalent-dependent changes in intrinsic neuronal excitability and tested if CaSR is transducing decreases in [Ca^2+^]_o_ into NALCN-mediated depolarizations to trigger action potentials (Lu et al., 2010). We found no evidence that this specific mechanism was active in neocortical neurons (Figure 2). Instead, we determined that the vast majority of divalent-dependent neuronal excitability was mediated via VGSCs in three ways. Decreasing the concentration of external divalents activated VGSCs at the resting membrane potential and depolarized the membrane toward the action potential threshold (Figure 6). This occurred because the decreased divalent concentration hyperpolarized the VGSC window current toward the membrane potential increasing sodium currents and the likelihood of action potential generation (Figure 3). Unexpectedly the deletion of CaSR modulated VGSC gating, decreasing the sensitivity of current activation to depolarization via an unidentified mechanism (Figure 3). Deletion of CaSR also indirectly affected action potential generation by modestly hyperpolarizing the membrane potential (Figure 1). While the actions of [Ca^2+^]_o_ on VGSCs were responsible for the vast majority of the [Ca^2+^]_o_-dependent neuronal excitability, using Gd^3+^ we isolated small divalent-dependent inward currents in about half of the neurons (Figure 5). These Gd^3+^-sensitive currents presumably reflected activation of NALCN, and were unaffected by CaSR deletion, but their relatively small size compared to TTX-sensitive divalent-dependent inward currents indicate that they would be minor contributors to divalent-dependent excitability compared to VGSCs (Figures 5 and 6).

The fractions of the divalent-dependent currents and depolarizations that were sensitive to TTX were surprisingly large compared to those that were Gd^3+^-sensitive (Figures 5D and 6C) indicating the relative importance of VGSC- and NALCN-mediated contributions to divalent-dependent excitability respectively. The resistance of NALCN to TTX (Lu et al., 2007; Swayne et al., 2009) reassures that the relatively large TTX-sensitive component is due to selective block of VGSC currents. Persistent subthreshold VGSC currents have been shown to determine spiking rates in other central neurons (Taddese and Bean, 2002; Gorelova and Seamans, 2015) and so the increased VGSC currents we observed in T_0.2_ are well-positioned to explain the increased action potential frequency (Figure 6). We are unable to determine from these experiments which neuronal compartment is most affected by the change in [Ca^2+^]_o_ (Gorelova and Seamans, 2015). However, the physiological impact of VGSC-mediated divalent-dependent excitability may be enormous overall because of the dynamic nature of [Ca^2+^]_o_in vivo where it decreases from basal (1.1–1.2 mM) by 30–80% (Nicholson et al., 1978; Ohta et al., 1997; Pietrobon and Moskowitz, 2014). The overall computational effects of physiological decrements in [Ca^2+^]_o_ will be complex because the increased action potential generation due to changes on VGSCs (Figures 3 and 6) will be confounded by the impact of reduced Ca^2+^ entry through VACCs (Hess et al., 1986; Weber et al., 2010; Williams et al., 2012), reduced excitatory synaptic transmission (Neher and Sakaba, 2008; Vyleta and Smith, 2011), and altered CaSR-mediated signaling at the nerve terminal (Phillips et al., 2008; Chen et al., 2010; Vyleta and Smith, 2011).

It remains unclear why NALCN was the dominant effector of divalent-dependent excitability in hippocampal (Lu et al., 2010) but not neocortical neurons (Figure 6). Could our use of [Ca^2+^]_o_ and [Mg^2+^]_o_ rather than [Ca^2+^]_o_ alone be responsible? We changed divalents simultaneously to provide a strong stimulus to CaSR-signaling and VGSC gating, both of which are sensitive to [Ca^2+^]_o_ and [Mg^2+^]_o_ (Frankenhaeuser and Hodgkin, 1957; Brown et al., 1993). Consequently, the same pathways were expected to respond to changes in divalents or [Ca^2+^]_o_ alone, since the potentially confounding effects on synaptic transmission were blocked in our experiments. Another difference is that we counted spontaneous action potentials as the main measure of excitability whereas others have focused on action potentials elicited by direct injection. We used spontaneous activity to allow us to isolate the depolarization (Figure 2) that was hypothesized to arise from NALCN activation and trigger action potentials following the reduction of external divalent concentration changes (Figure 2). Spontaneous and depolarization-elicited action potentials have been recognized as forms of [Ca^2+^]_o_-dependent excitability for >60 years (Frankenhaeuser and Hodgkin, 1957) and both types of activity were increased here when external divalent concentrations were decreased (Figure 1 and Figure 1—figure supplement 2). Because we observed increased excitability, despite the injection of a current to bring the steady state membrane potential back to that recorded in T_1.1_, mechanisms other than a voltage-independent non-selective cation channel, like NALCN, must have been active (Figure 1 and Figure 1—figure supplement 2). Similarly, the increased spikes elicited by transient current injections in low [Ca^2+^]_o_ in hippocampal neurons occurred after the steady state membrane potential was set to −80 mV using a longer current injection (Lu et al., 2010). The long injection would have reversed the NALCN-mediated depolarization in low [Ca^2+^]_o_ and so the mechanism by which the increased excitability occurred is unclear. One possible explanation is that at low [Ca^2+^]_o_ NALCN could have been further activated by shorter depolarizing current injections; however, this is at odds with the lack of voltage-dependence of NALCN (Lu et al., 2010). Could NALCN be operating via a different mechanism? One possibility is that NALCN activation is enhancing excitability measured at the soma by enhancing calcium entry into nerve terminals (directly or modifying the action potential waveform and VACC activation) and strengthening excitatory synaptic transmission onto the neuron under study. This would require that the enhancement of synaptic transmission by NALCN be greater than the reduction due to reduced Ca^2+^ entry (Neher and Sakaba, 2008) but could be addressed by recording directly from terminals (Ritzau-Jost et al., 2021) or by determining if NALCN deletion has the same effect after blocking glutamatergic transmission. However, the loss of NALCN could be contributing to [Ca^2+^]_o_-dependent changes in excitability independent of a depolarization based on other reports. A number of mechanisms have been postulated to explain how a persistent sodium leak into excitable cells at rest can affect excitability (Sokolov et al., 2007). Such mechanisms or other compensatory changes in neuronal function, as observed with null-mutant animals (Jun et al., 1999), could arise from the loss of NALCN and possibly contribute to the reduced sensitivity of hippocampal neurons to decreased [Ca^2+^]_o_ (Lu et al., 2010). Lastly, the apparent difference between the studies could reflect different properties of hippocampal and neocortical neurons. While possible it still remains unclear why the deletion of NALCN or UNC-79 completely ablated [Ca^2+^]_o_-dependent excitability in hippocampal neurons (Lu et al., 2010) since these neurons contain VGSCs that retain sensitivity to changes in [Ca^2+^]_o_ (Isaev et al., 2012). However, if the UNC79-UNC80-NALCN pathway modulates VGSC function this could explain how loss of NALCN or UNC-79 could delete acute divalent-dependent changes in VGSC function and excitability. NALCN appears to transduce [Ca^2+^]_o_- and G-protein-dependent excitability in other neurons (Philippart and Khaliq, 2018) but GPCRs other than CaSR may be involved (Kubo et al., 1998; Tabata and Kano, 2004) and under certain conditions Ca^2+^ directly blocks NALCN (Chua et al., 2020). Further characterization of the UNC79-UNC80-NALCN signaling pathway is essential given the major changes in neurological function that have been described following mutations of NALCN or upstream co-molecules such as UNC79 and UNC80 (Stray-Pedersen et al., 2016; Bourque et al., 2018; Kuptanon et al., 2019).

In a small fraction of the neocortical neurons (Figures 5 and 6) there was a modest inward current or depolarization with the lowering of extracellular divalent concentration once VGSCs had been blocked. In a few cases, they were sensitive to 10 µM Gd^3+^ consistent with a NALCN-mediated effect and those that were resistant were consistent with other divalent-dependent non-selective cation channels (Ma et al., 2012b). However, deletion of CaSR did not decrease divalent-dependent depolarizations and after membrane potential matching did not impact divalent-dependent excitability (Figures 2E and 6B). While CaSR-NALCN signaling did not contribute to divalent-dependent excitability in neocortical neurons (Figures 2 and 6) it was clear that Casr^-/-^ neurons were substantially less sensitive to changes [Ca^2+^]_o_ (Figure 1A,B). The reduced [Ca^2+^]_o_ sensitivity in these neurons is attributable to the combination of altered VGSC gating (Figure 3E,F) and the hyperpolarized RMP (Figure 1E). Although CaSR did not affect the amplitude of the shift in V_0.5_ following the switch to T_0.2_, the gating characteristics of VGSC activation was depolarized by loss of CaSR (Figure 3EF). Could CaSR stimulation activate G-proteins and regulate the V_0.5_ for VGSC currents (Figure 3E,F)? In neocortical neurons, G-protein activation hyperpolarized VGSC gating and this was blocked by GDPβS (Mattheisen et al., 2018) which is inconsistent with the effect we observed here. Other possible explanations are that CaSR could regulate VGSC subunit expression or post translational modification (Cantrell et al., 1996; Zhang et al., 2019), and this may represent a compensatory mechanism similar to that observed with other mutant mouse models (Jun et al., 1999). Loss of CaSR also hyperpolarized the neocortical neurons (Figure 1I) and this may have been due to decreased function of depolarizing components or stimulation of hyperpolarizing elements. There are a number of candidate channels and pumps that have been shown to regulate the RMP in cortical neurons (Tavalin et al., 1997; Talley et al., 2001; Bean, 2007; Harnett et al., 2015; Hu and Bean, 2018). The changes in VGSC gating and RMP in *Casr^-/-^* neurons may be attributable to homeostatic mechanisms that compensate for perturbations in network activity and have been observed in central and peripheral neurons (Turrigiano, 2008).

Overall, our studies indicate that divalent-dependent excitability in neurons is largely attributable to actions of extracellular calcium on the VGSC function. Given the dynamic nature of brain extracellular calcium, this mechanism is likely to impact neuronal signaling greatly under physiological and pathological conditions. CaSR-dependent reduction of VGSC sensitivity to membrane potential adds further complexity to extracellular calcium signaling and identifies another potential mechanism by which CaSR stimulation may influence neuronal death following stroke and traumatic brain injury (Kim et al., 2013; Hannan et al., 2018).

## Materials and methods

**Key resources table:** 

Reagent type (species) or resource	Designation	Source or reference	Identifiers	Additional information
Gene (*M. musculus*)	Casr	GenBank	Casr	
Strain, strain background (*M. musculus*)	Mouse wild-type strain C57BL/6J × 129×1	The Jackson Laboratory	RRID:MGI:5652742	
Genetic reagent, strain background (*M. musculus*)	Mouse expressing Nestin-cre mutation	The Jackson Laboratory as used in Sun et al., 2018	Stock No. 003771	C57/BL6J and 129S4 background strain
Genetic reagent, strain background (*M. musculus*)	Mouse with Lox mutation to delete exon 7 of Casr	Laboratory of Dr. Wenhan Chang, UCSF (Chang et al., 2008)	*Casr*^fl/fl^	C57/BL6J and 129S4 background strain
Sequence-based reagent	Casr	Applied Biosystems	Mm00443377_m1	Quantitative PCR Mouse probe set
Sequence-based reagent	Actb	Applied Biosystems	Mm04394036_g1	Quantitative PCR Mouse probe set
Sequence-based reagent	Nes-Cre1 primer	IDT	GCAAAACAGGCTCTAGCGTTCG	
Sequence-based reagent	Nes-Cre2 primer	IDT	CTGTTTCACTATCCAGGTTACGG	
Sequence-based reagent	P3U primer	IDT	TGTGACGGAAAACATACTGC	
Sequence-based reagent	Lox R primer	IDT	GCGTTTTTAGAGGGAAGCAG	

### Genotyping and CaSR mutant mice

ConWT animals were obtained from an established colony consisting of a stable strain of C57BL/6J and 129 × 1 mice. The *Casr^-/-^* mice were generated by breeding floxed Casr (Chang et al., 2008) and nestin Cre mice (B6.Cg-Tg (Nes-cre)1Kln/J, The Jackson Laboratory) as described previously (Sun et al., 2018). The lox sites were positioned to delete Casr exon seven which resulted in the loss of Casr expression (Chang et al., 2008) and the nestin promoter was designed to ensure floxing occurred in neuronal and glial precursors. The *Nes^Cre^* mice were generated by crossing mice that did not contain the flox Casr mutation but did express the nestin Cre mutation. The *Casr^-/-^* and *Nes^Cre^* mice were all generated using a background C57BL/6J and 129S4 strain. Tail DNA extraction was performed using the Hot Shot Technique with a 1–2 hr boil (Montero-Pau et al., 2008). The presence or absence of the flox Casr mutation and Cre mutation were confirmed by PCR for each mouse. MoPrimers used for cre PCR were: Nes-cre1: GCAAAACAGGCTCTAGCGTTCG, Nes-cre2: CTGTTTCACTATCCAGGTTACGG; run on a 1% agarose gel. Primers for lox PCR were: P3U: TGTGACGGAAAACATACTGC, Lox R: GCGTTTTTAGAGGGAAGCAG; run on a 1.5% agarose gel (Chang et al., 2008). Successful deletion of Casr in the neocortical cultures was confirmed by measuring mRNA expression levels with the QuantStudio12K Flex Real-time PCR System (Applied Biosystems) and the TaqMan mouse probe set to Casr (Mm00443377_m1) with ActB (Mm04394036_g1) as the endogenous control (Figure 1—figure supplement 1). The paper describes experiments comparing the effects of CaSR deletion using the *Casr^-/-^* mice. After confirming that conWT and *Nes^Cre^* neurons responded similarly to changing external divalents (Figure 1A–D) we used *Nes^Cre^* neurons and *Casr^-/-^* neurons to examine if Casr was responsible for the sensitivity to extracellular divalents. This comparison avoided possible confounding cre-dependent effects (Qiu et al., 2011). In later experiments, we used conventional WT to ensure that our measurements of the relative size of the effect of VGSC and NALCN were not impacted by cre-dependent effects (Qiu et al., 2011).

### Neuronal cell culture

Neocortical neurons were isolated from postnatal day 1–2 mouse pups of either sex as described previously (Phillips et al., 2008). All animal procedures were approved by V.A. Portland Health Care System Institutional Animal Care and Use Committee in accordance with the U.S. Public Health Service Policy on Humane Care and Use of Laboratory Animals and the National Institutes of Health Guide for the Care and Use of Laboratory Animals. The active protocols covering this work are 4254–19 and 4359–20. Animals were decapitated following induction of general anesthesia with isoflurane and then the cerebral cortices were removed. Cortices were incubated in trypsin and DNase and then dissociated with a heat polished pipette. Dissociated cells were cultured in MEM plus 5% FBS on glass coverslips. Cytosine arabinoside (4 µM) was added 48 hr after plating to limit glial division. Cells were used, unless otherwise stated after ≥14 days in culture.

### Electrophysiological recordings

Cells were visualized with a Zeiss IM 35 inverted microscope. Whole-cell voltage-and current-clamp recordings were made from cultured neocortical neurons using a HEKA EPC10 amplifier. Except where stated in the text, extracellular Tyrodes solution contained (mM) 150 NaCl, 4 KCl, 10 HEPES, 10 glucose, 1.1 MgCl_2_, 1.1 CaCl_2_, pH 7.35 with NaOH. Calcium and magnesium concentrations were modified as described in the Figure legends. The CaSR and surface charge screening are both sensitive to Ca^2+^ and Mg^2+^ with Ca^2+^ being two to three times more effective in both processes (Frankenhaeuser and Hodgkin, 1957; Brown et al., 1993). We modified the divalent concentrations simultaneously to utilize a greater fraction of the dynamic range of the phenomenon under study and to avoid irreversible changes that can occur in Ca^2+^-free solutions (Frankenhaeuser and Hodgkin, 1957). Synaptic transmission was blocked by the addition of (in µM) 10 CNQX, 10 Gabazine, and 50 APV to the bath solution. Most recordings were made using a potassium gluconate intracellular solution containing (mM) 135 K-gluconate, 10 HEPES, 4 MgCl_2_, 4 NaATP, 0.3 NaGTP, 10 phosphocreatine disodium, pH 7.2 with KOH hydroxide. In nucleated patch experiments, the pipette solution contained (in mM) 113 Cesium methane sulfonate, 1.8 EGTA, 10 HEPES, 4 MgCl_2_, 0.2 CaCl_2,_ 4 NaATP, 0.3 NaGTP, 14 phosphocreatine disodium, pH 7.2 with TEA hydroxide. Electrodes used for recording had resistances ranging from 2 to 7 MΩ. Voltages have been corrected for calculated liquid junction potentials (JPCalc, Professor P. H. Barry) and were 14 or 15 mV for all recordings. All experiments were performed at room temperature (21–23°C).

### Data acquisition and analysis

Whole-cell voltage-and current-clamp recordings were made using a HEKA EPC10 USB amplifier, filtered at 2.9 kHz using a Bessel filter, and sampled at 20 kHz during injection protocols and 10 kHz during continuous acquisition. Analysis was performed using Igor Pro (Wavemetrics, Lake Oswego, OR) and Minianalysis (Synaptosoft). Data values are reported as mean ± SEM. Statistical tests were performed using GraphPad Prism (6) and p-values<0.05, 0.01, 0.001, and 0.0001 were indicate with *, **, ***, and ****. All post-hoc tests were Sidak compensated for multiple comparisons. Data were log-transformed to improve normalization in Figure 2D. To ensure non-zero values, minimize bias, and allow logarithmic transformation, each action potential frequency measurement was increased by 0.02 as the duration of the T_1.1_ recording at −70 mV was 50 s.

### Solution application

Solutions were applied by gravity from a glass capillary (1.2 mm outer diameter) placed ~1 mm from the neuron under study. Solutions were switched manually using a low dead volume manifold upstream of the glass capillary. CNQX and Gabazine were supplied by Abcam. KB-R7943 Mesylate was supplied by Tocris. Creatine Phosphate was supplied by Santa Cruz Biotech. Cinacalcet was supplied by Toronto Research Chemicals and Tetrodotoxin by Alomone Other reagents were obtained from Sigma-Aldrich.

## Data Availability

All data generated are in the manuscript and supporting files. Source provided for Figures 1, 2, and 6 in the manuscript.

## Additional files

Transparent reporting form
